# Enhanced Organic Electrochemical Transistor Performance of Donor–Acceptor Conjugated Polymers Modified with Hybrid Glycol/Ionic Side Chains by Postpolymerization Modification

**DOI:** 10.1021/acs.chemmater.3c00327

**Published:** 2023-04-11

**Authors:** Bowen Ding, Il-Young Jo, Hang Yu, Ji Hwan Kim, Adam V. Marsh, Edgar Gutiérrez-Fernández, Nicolás Ramos, Charlotte L. Rapley, Martina Rimmele, Qiao He, Jaime Martín, Nicola Gasparini, Jenny Nelson, Myung-Han Yoon, Martin Heeney

**Affiliations:** † Department of Chemistry and Centre for Processable Electronics, Imperial College London, Molecular Sciences Research Hub (White City Campus), 80 Wood Lane Shepherd’s Bush, London W12 0BZ, United Kingdom; ‡ School of Materials Science and Engineering, Gwangju Institute of Science and Technology, 123 Cheomdangwagi-ro, Buk-gu, Gwangju 61005, Republic of Korea; § Department of Physics and Centre for Processable Electronics, Imperial College London, South Kensington Campus, London SW7 2AZ, United Kingdom; ∥ KAUST Solar Center, Physical Sciences and Engineering Division (PSE), King Abdullah University of Science and Technology (KAUST), Thuwal 23955-6900, Saudi Arabia; ⊥ POLYMAT University of the Basque Country UPV/EHU, Manuel de Lardizabal 3, 20018 Donostia-San Sebastián, Spain; # Grupo de Polímeros, Departamento de Física e Ciencias da Terra, Centro de Investigacións Tecnolóxicas (CIT), Universidade da Coruña, Esteiro, 15471 Ferrol, Spain

## Abstract

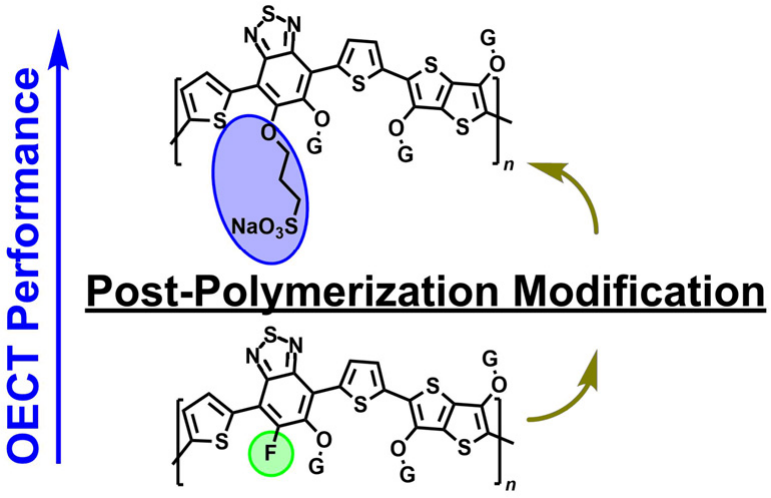

Emergent bioelectronic technologies are underpinned by the organic electrochemical transistor (OECT), which employs an electrolyte medium to modulate the conductivity of its organic semiconductor channel. Here we utilize postpolymerization modification (PPM) on a conjugated polymer backbone to directly introduce glycolated or anionic side chains via fluoride displacement. The resulting polymers demonstrated increased volumetric capacitances, with subdued swelling, compared to their parent polymer in *p*-type enhancement mode OECTs. This increase in capacitance was attributed to their modified side chain configurations enabling cationic charge compensation for thin film electrochemical oxidation, as deduced from electrochemical quartz crystal microbalance measurements. An overall improvement in OECT performance was recorded for the hybrid glycol/ionic polymer compared to the parent, owing to its low swelling and bimodal crystalline orientation as imaged by grazing-incidence wide-angle X-ray scattering, enabling its high charge mobility at 1.02 cm^2^·V^–1^·s^–1^. Compromised device performance was recorded for the fully glycolated derivative compared to the parent, which was linked to its limited face-on stacking, which hindered OECT charge mobility at 0.26 cm^2^·V^–1^·s^–1^, despite its high capacitance. These results highlight the effectiveness of anionic side chain attachment by PPM as a means of increasing the volumetric capacitance of *p*-type conjugated polymers for OECTs, while retaining solid-state macromolecular properties that facilitate hole transport.

## Introduction

Organic electrochemical transistors (OECTs) are an emergent platform of devices that currently dominate bioelectronics research.^1−3^ The amplification capabilities of OECTs enable their use as biosensors,^4^ while the ability to mimic short- and long-term neural plasticity set forth their implementation in neuromorphic computing.^5−7^ OECTs also inspire other bioelectronic devices, such as the organic electrochemical diode.^8^ Unlike organic field-effect transistors, electrochemical gating of OECTs via an electrolyte necessitates the volumetric penetration of counterions to achieve modulation of charge carrying polarons/bipolarons throughout the bulk of the channel material.^9,10^ Correspondingly, OECT channels between the source and the drain electrodes require the application of organic mixed ionic-electronic conductors (OMIECs), which are typically conjugated polymers featuring oligomeric glycol or ionic side chains that enable ionic movements about an electronically conductive conjugated backbone.^11,12^ Contemporary OECT research favors the development of enhancement mode devices,^13^ where the conductivity of the OMIEC channel material is negligible at resting gate potential, and grows with increased gate bias.

To date, Bernards and Malliaras’ model prevails in describing OECT operation, which applies separate ionic and electronic circuits to simulate their concurrent ionic and electronic charge movement during device operation.^14^ The transconductance of an OECT, a key figure-of-merit, is derived from this model and is proportional to the product of electronic charge mobility and volumetric capacitance of the channel material.^15^ Although theoretical orthogonality is assumed, in reality there is a strong mutual interplay between ionic and electronic charge conduction in an OECT, complicating the OMIEC material design optimization process.^16,17^ This interplay is mostly defined by the morphological changes to the active layer upon injection of solvated counterions driven by gate biases, which disturb electronic charge conduction pathways.^18−21^ Transconductance optimization therefore requires increasing the ability of thin films to accommodate ions, while maintaining a favorable microstructure for electronic charge transport, not least by minimizing consequential swelling, which can be achieved through the side chain engineering of OMIECs.^22,23^

The side chain abundance and distribution of glycolated OMIECs has been demonstrated to disproportionately affect their OECT performance.^24−29^ At low and/or uneven loadings of glycol side chains, thin film ability to accommodate ions is limited, lowering volumetric capacitance.^11^ On the other hand, long glycol side chains, for example, hexaethylene glycol side chains, can form domains that promote excessive solvent (water) uptake, according to molecular dynamics simulations, which disconnects electronic charge conduction pathways.^24^ The incorporation of ionic side chains can promote ion transport, but conjugated polyelectrolytes with large ionic components can be water-soluble, requiring the use of performance-diminishing cross-linkers during OECT fabrication.^30−32^ The inclusion of alkyl side chains reduces the water solubility and swelling of conjugated polyelectrolytes^33^ but undesirably hinders ion transport.^17^ Although OMIECs featuring hybrid alkyl/glycol^28,34,35^ and alkyl/ionic side chains^33^ have been studied to tune the balance between capacitance and charge mobility, there has been limited exploration of hybrid glycol/ionic side chains in OMIECs to optimize their OECT performance by raising volumetric capacitance without compromising electronic charge mobility, possibly due to the challenging purification of ionic monomeric precursors.

We hypothesized that the enhancement mode OECT performance of **PgBT(F)2gTT**,^36^ a state-of-the-art donor–acceptor (D-A) *p*-type polymer, could be optimized by incorporating a minor component of anionic side chains with mobile cations, which would encourage cation dynamics in its charging mechanism.^37^ Concurrent charge balancing of hole injection with anion inclusion and cation expulsion may improve material ion transport and volumetric capacitance, with lower total active swelling. By taking advantage of the postpolymerization modification (PPM) by nucleophilic aromatic substitution (S_N_Ar) of fluorine groups on this polymer, we can directly append anionic side chains onto the polymer, circumventing the inhibitive precursor purification issues inherent to a bottom-up synthetic approach to such materials.^38−40^ Moreover, the use of alcohol functionalized sulfonates would result in aryl-ether side chain linkage formation, maintaining a procession of S···O interactions along the backbone. This is predicted to preserve backbone planarity, as confirmed by DFT studies (Figures S1–S4), which is crucial for charge transport and accessible electrochemical oxidation.^41−43^ We report the development of **PgBT(Ion)2gTT**, a **PgBT(F)2gTT** derivative featuring mixed glycol/anionic sulfonate side chains. Its OECT performance was found to be superior to that of **PgBT(F)2gTT** and similarly synthesized **PgBT(TriEG)2gTT** (with all glycol side chains), demonstrating that the hybrid glycol/ionic side chain configuration is effective in increasing material capacitance without causing an electronic charge transport trade-off.

## Results and Discussion

**Scheme 1:**
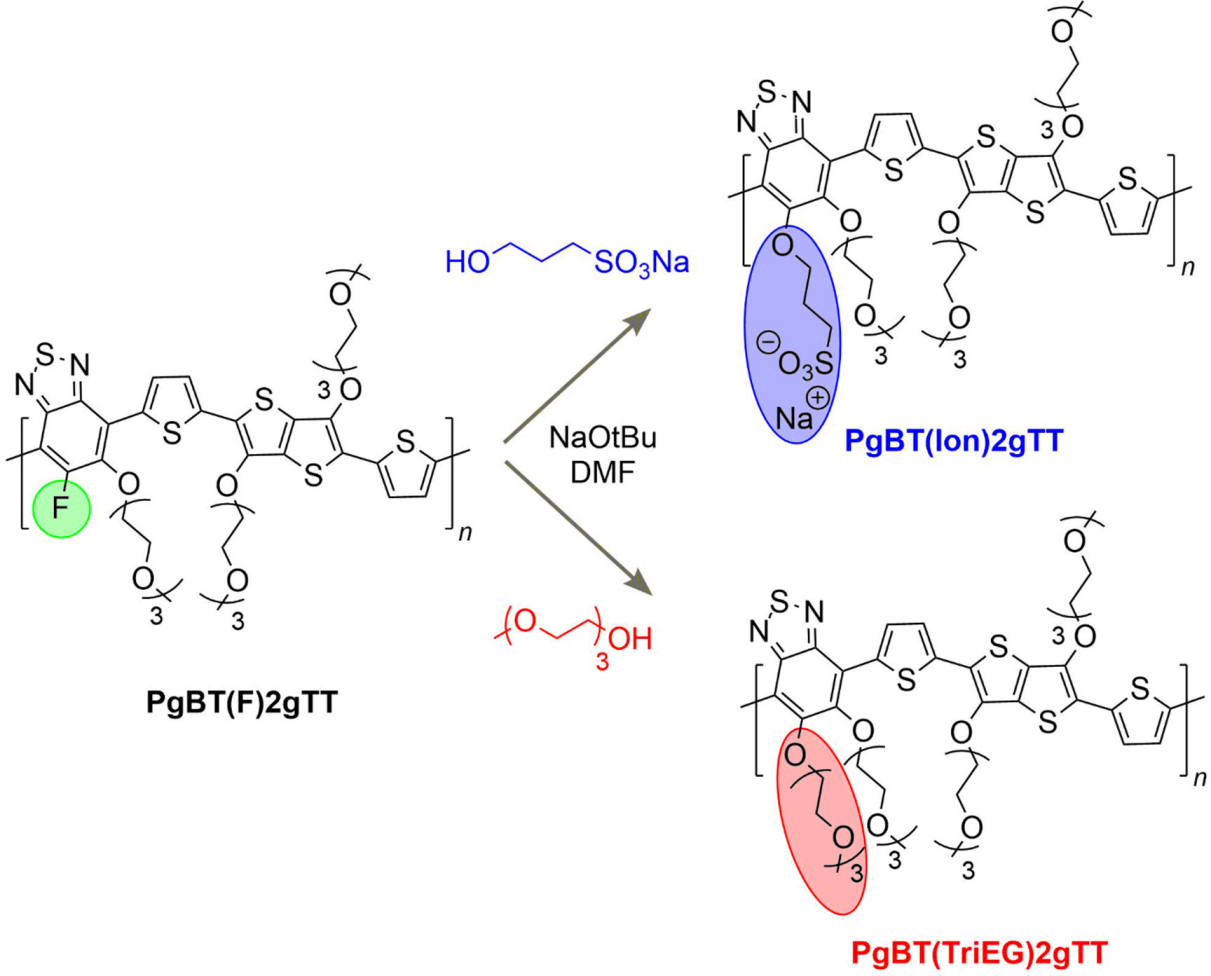
PPM Conversion of **PgBT(F)2gTT** to **PgBT(Ion)2gTT** and **PgBT(TriEG)2gTT**

Functionalization of **PgBT(F)2gTT** with sodium 3-hydroxypropane-1-sulfonate or triethylene glycol monomethyl ether in the presence of NaO*t*Bu in DMF at 120 °C was conducted to give **PgBT(Ion)2gTT** and **PgBT(TriEG)2gTT**, respectively (Scheme 1). Following precipitation and solvent washing to remove unreacted excess nucleophile and NaO*t*Bu, both polymers were obtained in excellent yield (>80%).

The structures of **PgBT(Ion)2gTT** and **PgBT(TriEG)2gTT** were confirmed by ^1^H and ^19^F NMR (Figures S5–S8), as well as through the fragmental analysis of their MALDI-ToF traces (Figures S9 and S10). Complete PPM conversion was confirmed for both derivatives through the disappearance of all ^19^F NMR signals, coupled with defined changes to fragment sizes observed by MALDI-ToF. In contrast, we observed little change to their FTIR spectra (Figure S11) upon PPM, which can be attributed to the overpowering presence of signals originating from glycol chains, coupled with the conformational similarity between parent and derivatives. The thermal properties of both **PgBT(Ion)2gTT** and **PgBT(TriEG)2gTT** were probed by differential scanning calorimetry (Figures S12 and S13), which revealed no thermal processes between 50–300 °C, much like parent **PgBT(F)2gTT**.^36^ GPC analysis of macromolecular properties was complicated by polymer aggregation, as is observed with many examples of glycolated polymers,^36^ but together with MALDI confirmed the retention of molecular weight distribution upon conversion of **PgBT(F)2gTT** to **PgBT(Ion)2gTT** and **PgBT(TriEG)2gTT** (Figures S9, S10, S14, and S15).

The CHCl_3_ solution state UV/vis absorption spectra of **PgBT(TriEG)2gTT** and **PgBT(Ion)2gTT** show similar absorption onsets at 770 nm (Figures S16 and S17) but noticeably different spectral shapes. The differing spectral features are attributable to varying degrees of solution aggregation that is more pronounced for **PgBT(Ion)2gTT**. The S0–S1 transition of **PgBT(Ion)2gTT** features a broad absorption from 600–700 nm, whereas a distinct absorption at λ_max_ = 606 nm is observed for **PgBT(TriEG)2gTT**, with a longer wavelength shoulder at 670 nm. The spectra for both polymers are red-shifted and broadened in the solid state relative to solution (Figures S18 and S19), as a result of planarization and packing.

**Figure 1:**
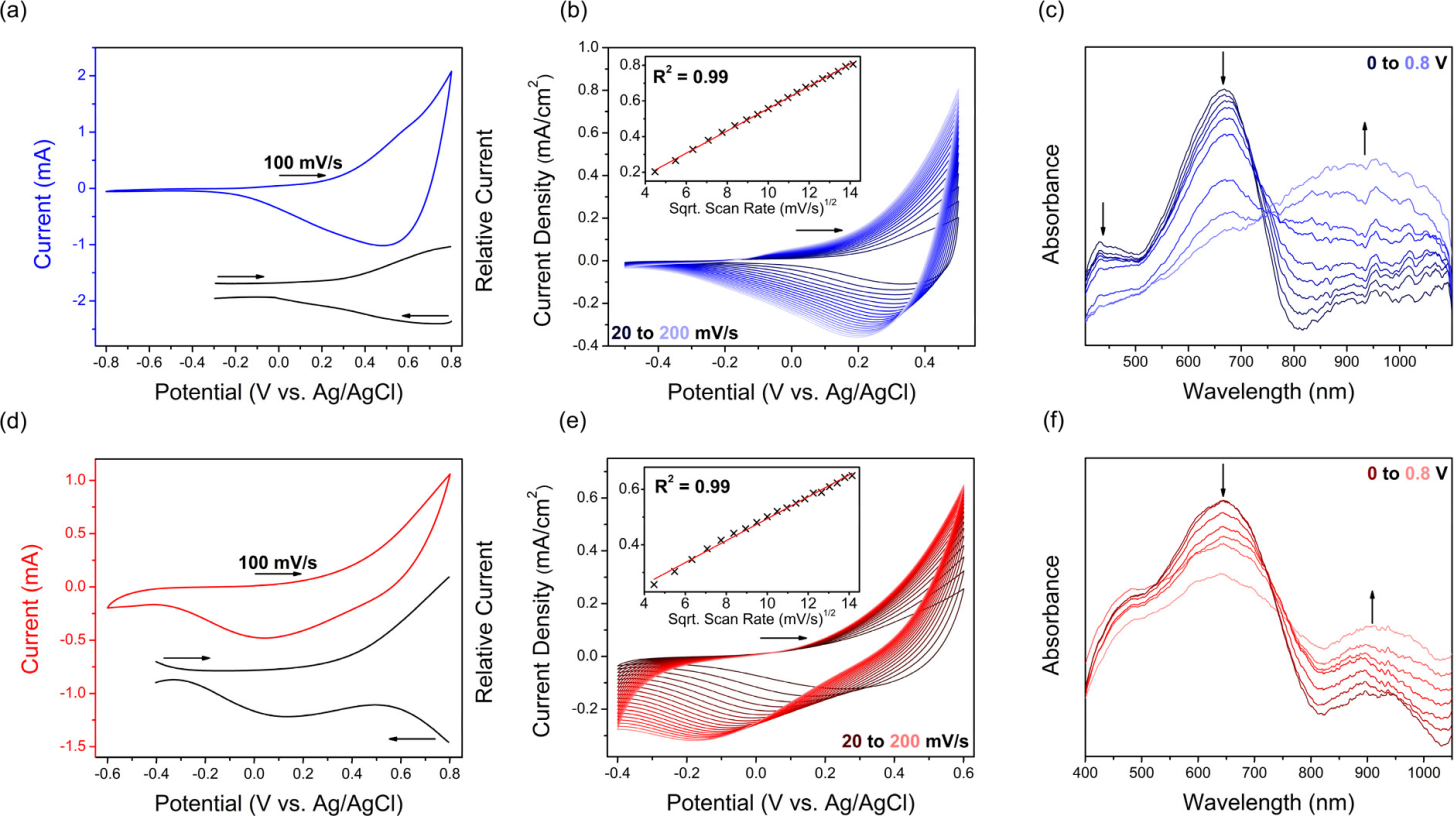
Thin film electrochemistry in 0.1 M KCl/H_2_O of **PgBT(Ion)2gTT** (top, blue) and **PgBT(TriEG)2gTT** (bottom, red) showing (a/d) CV and SQW (black); (b/e) scan rate dependence of CV, with inset plot of peak currents at 0.5/0.6 V vs Ag/AgCl (respectively) against the square root of scan rate (linear regression in red); and (c/f) UV/vis SEC with spectral changes at 0.1 V intervals. Arrows indicate scan directions and spectral progression.

The DFT calculated HOMO of the **PgBT(Ion)2gTT** and **PgBT(TriEG)2gTT** backbone lies at −4.57 eV, which is slightly shallower than that of **PgBT(F)2gTT** (−4.65 eV), as a result of replacing each electron-withdrawing fluorine with an electron-donating alkoxy group. This difference is reflected in their thin film cyclic voltammetry (CV) and square wave voltammetry (SQW) electrochemical data in 0.1 M KCl/H_2_O (Figure 1a,d), where an onset of oxidation at 0.2 V vs Ag/AgCl (HOMO = −4.8 eV) was observed for both derivative polymers (*cf*. 0.3 V/–4.9 eV for **PgBT(F)2gTT**). Scan rate dependence CV data was also collected for **PgBT(Ion)2gTT** and **PgBT(TriEG)2gTT** (Figure 1b,e), revealing a linear correlation between oxidative currents and the square root of corresponding scan rates for both materials, evidencing diffusion limited electrochemical oxidation with volumetric penetration of counterions.^44^ Cycling CV data demonstrated the excellent electrochemical stability of **PgBT(Ion)2gTT** and **PgBT(TriEG)2gTT** (Figures S22 and S23). The thin film electrochemistry of **PgBT(Ion)2gT** and **PgBT(TriEG)2gTT** in 0.1 M [*n*-Bu_4_N]PF_6_/MeCN demonstrated behavior similar to the aqueous electrolyte data (Figures S24–S31).

To confirm the reversible generation of charge-carrying polarons upon electrochemical oxidation, thin films of **PgBT(Ion)2gTT** and **PgBT(TriEG)2gTT** were probed by UV/vis spectroelectrochemistry (SEC). Upon application of an anodic potential of 0.2 V, which had increments of 0.1 V steps up to 0.8 V, a gradual quenching of **PgBT(Ion)2gTT** and **PgBT(TriEG)2gTT** ground state transitions was observed, concurrent with the appearance and intensification of broad polaron bands centered around 950 and 900 nm, respectively (Figure 1c,f). Upon spectral changes peaking at 0.8 V, the applied potential was returned to 0 V, restoring ground state spectra for both polymers (Figures S32 and S33), further confirming their excellent electrochemical reversibility.

**Figure 2:**
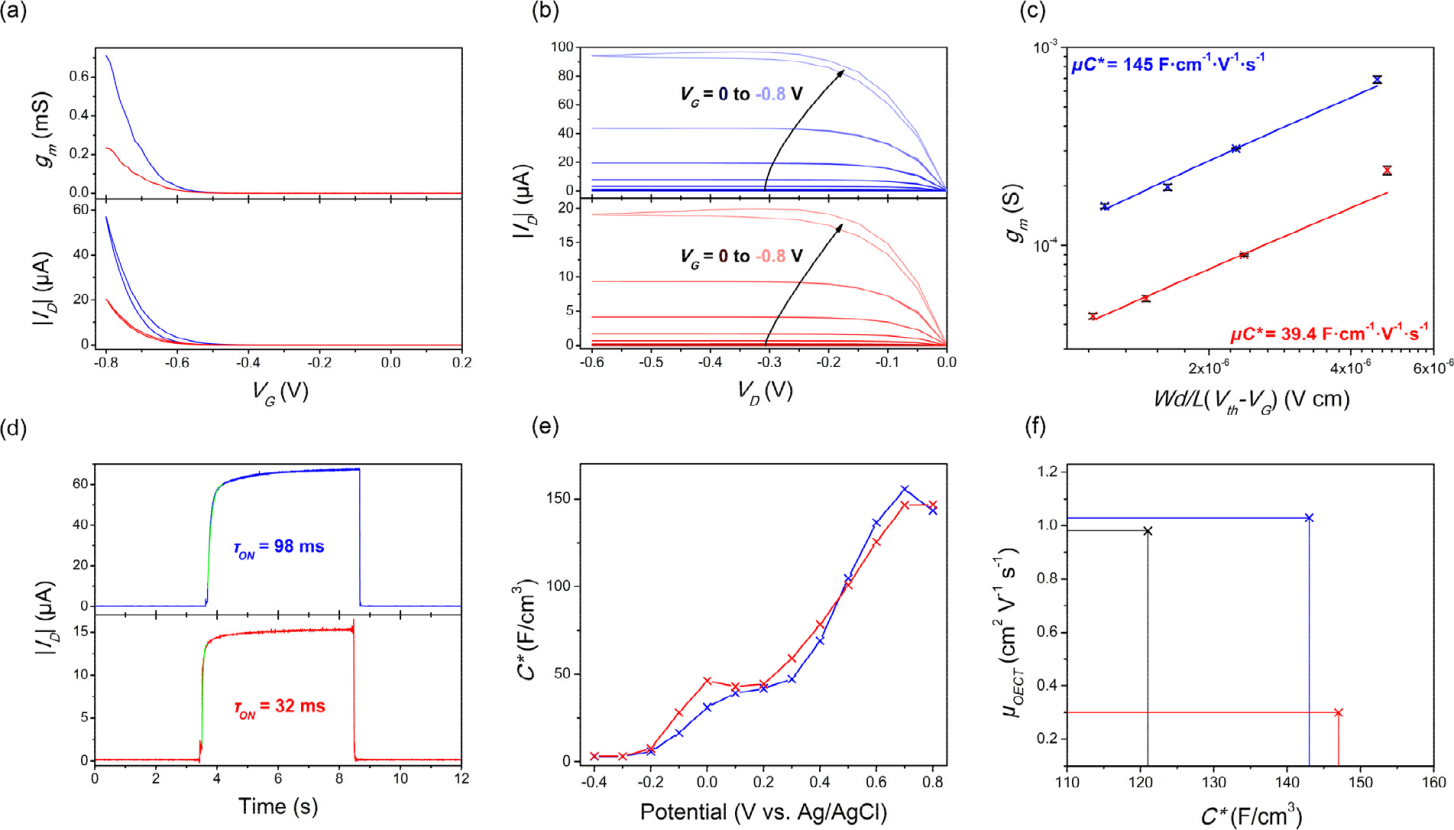
OECT performance of **PgBT(Ion)2gTT** (blue) and **PgBT(TriEG)2gTT** (red) showing (a) transconductance (top) and transfer curves (bottom) at *V*_*D*_ = −0.60 V; (b) output curves at stepped *V*_*G*_ in 0.05 V intervals (arrows indicate data at increased *V*_*G*_); (c) μC* extraction plot of transconductance against channel dimensions and operational parameters; (d) transient response (exponential decay function to obtain τ_*on*_ in green); (e) volumetric capacitances measured by EIS; and (f) plot of volumetric capacitances against OECT mobilities for both polymers as well as parent **PgBT(F)2gTT** (black).

The *p*-type enhancement mode OECT performances of **PgBT(Ion)2gTT** and **PgBT(TriEG)2gTT** were interpreted using the transconductance expression (eq 1, Tables 1 and S1, and Figures 2 and S34–S45; *V*_*G*_ = gate voltage, *I*_*D*_ = drain current).^9^ Compared to the volumetric capacitance of parent **PgBT(F)2gTT** (121 F·cm^–3^), both **PgBT(Ion)2gTT** and **PgBT(TriEG)2gTT** attained higher values of 143 and 147 F·cm^–3^, respectively, at 0.8 V vs Ag/AgCl. The charge mobility of **PgBT(Ion)2gTT** devices (1.02 cm^2^·V^–1^·s^–1^) calculated from device data at *V*_*G*_ = 0.8 V was similar to that of parent **PgBT(F)2gTT** (1.03 cm^2^·V^–1^·s^–1^), whereas the mobility of **PgBT(TriEG)2gTT** was significantly reduced (0.26 cm^2^·V^–1^·s^–1^) at *V*_*G*_ = 0.8 V. Overall, an improvement in OECT performance was observed for **PgBT(Ion)2gTT** over its parent **PgBT(F)2gTT**, with its μC* figure-of-merit at 145 F·cm^–1^·V^–1^·s^–1^, but a reduction was clear for **PgBT(TriEG)2gTT** devices, which achieved a lower μC* of 39.4 F·cm^–1^·V^–1^·s^–1^ owing to its diminished charge mobility. Normalized peak transconductances of **PgBT(F)2gTT**, **PgBT(Ion)2gTT**, and **PgBT(TriEG)2gTT** OECTs are compared with those of other recently reported D–A *p*-type OMIECs in Table S1.
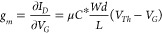
1

**Table 1:** OECT Performance Metrics, Averaged from at Least Five Films Per Material^a^

Material	*d*^b^ (nm)	*V*_*Th*_^c^ (V)	*I*_*ON*_/*I*_*OFF*_^d^	*g*_*m*_^e^ (mS)	*g*_*m*,*norm*_^f^ (S·cm^–1^)	μC*(F·cm^–1^·V^–1^·s^–1^)	C*^g^ (F·cm^–3^, EIS)	μ^h^ (cm^2^·V^–1^·s^–1^)	τ_*on*_^i^ (ms)
**PgBT(Ion)2gTT**	61.31 ± 3.64	–0.61	10^4^	0.69 ± 0.02	28.13	145.33	143	1.02	98
**PgBT(TriEG)2gTT**	59.72 ± 4.21	–0.60	10^3^	0.24 ± 0.01	10.04	39.37	147	0.26	32
**PgBT(F)2gTT**^j^	60.47 ± 5.50	–0.57	10^4^	0.59 ± 0.08	24.39	125.43	121	1.03	129

a Width/length (*W*/*L*) of channels were 80/20 μm for all devices.

b Channel thickness.

c Threshold voltage.

d ON/OFF ratio.

e Peak transconductance at *V*_*g*_ = −0.8 V.

f Peak transconductance normalized by channel geometry (*W·d·L*^–1^).

g Volumetric capacitance at 0.8 V vs Ag/AgCl; measured by electrochemical impedance spectroscopy (EIS, using a conventional 3 electrode system).

h Charge mobility calculated from figure-of-merit (μC*) and volumetric capacitance (C*).

i Transient response.

j **PgBT(F)2gTT** OECT performance remeasured using the current device configuration.

Upon modification of **PgBT(F)2gTT** to **PgBT(Ion)2gTT** and **PgBT(TriEG)2gTT**, transient responses of OECTs were improved, from a turn-on response of 129 ms to 98 and 32 ms, respectively. Note the slightly diminished switching speeds of our devices (*cf*. OMIECs of similar transconductances) can be attributed to active layer geometry and do not affect the comparative conclusions drawn.^45,46^ The long-term OECT cycling stabilities (between *V*_*G*_ = 0/–0.60 V, at *V*_*D*_ = −0.60 V) of **PgBT(Ion)2gTT** and **PgBT(TriEG)2gTT** were also excellent, with both materials retaining 60% of their initial drain currents after 100 and 75 min, respectively. However, cycling stabilities of **PgBT(Ion)2gTT** and **PgBT(TriEG)2gTT** were slightly compromised in comparison to parent **PgBT(F)2gTT**, owing to their shallower HOMOs, which make them more susceptible to ambient auto-oxidation reactions.

**Figure 3:**
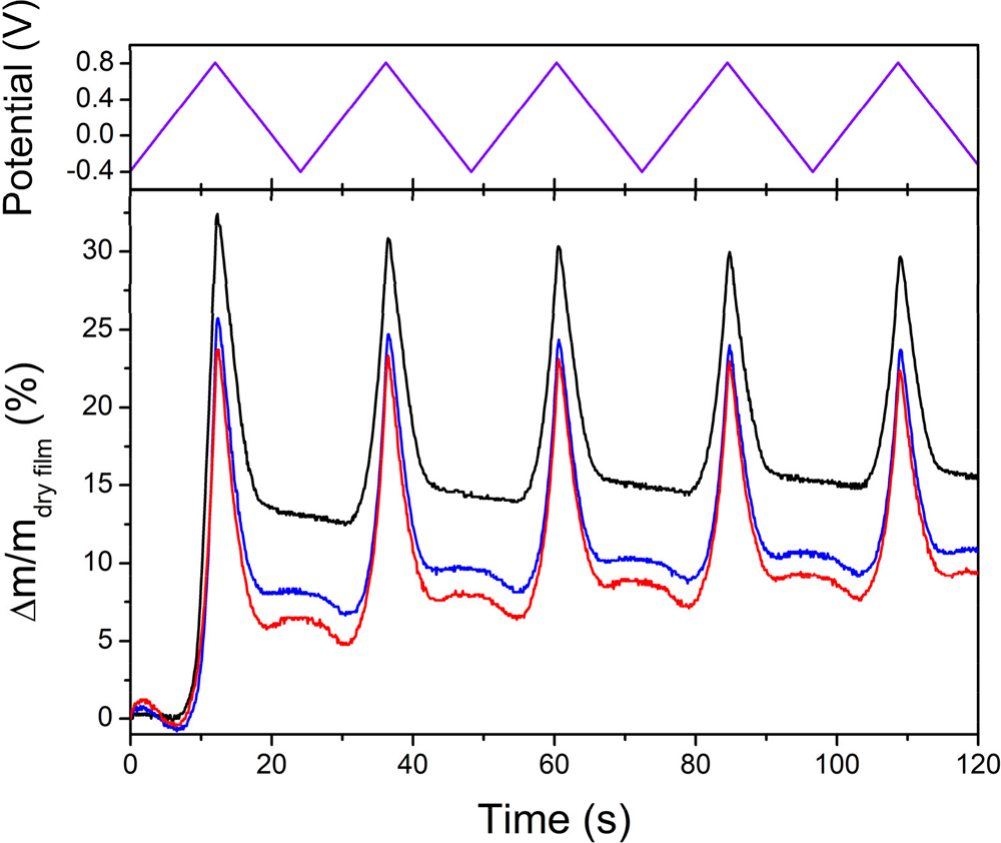
Fractional mass change calculated from EQCM measurements of **PgBT(Ion)2gTT** (blue) and **PgBT(TriEG)2gTT** (red), as compared to parent **PgBT(F)2gTT** (black) in 0.1 M KCl/H_2_O, with five cycles of potential applied between −0.4 to 0.8 V vs Ag/AgCl shown at the top (violet).

In order to investigate why both **PgBT(Ion)2gTT** and **PgBT(TriEG)2gTT** show increased volumetric capacitances compared to parent **PgBT(F)2gTT**, their swelling characteristics upon electrochemical biasing in 0.1 M KCl/H_2_O were probed using an electrochemical quartz crystal microbalance (EQCM, Figure 3). EQCM data for all films were collected over five cycles of applied potential between −0.4 and 0.8 V vs Ag/AgCl, at a scan rate of 100 mV/s. Two general observations were made from the EQCM data. First, at anodic potentials above their onsets of oxidation up to 0.8 V, a spike in mass uptake was observed for all three materials, correlated to anion injection for charge balancing the electronically injected holes. Mass uptakes for parent **PgBT(F)2gTT** at 0.8 V stabilized at *ca*. 30% from the third scan, which was higher than for derivatives **PgBT(Ion)2gTT** and **PgBT(TriEG)2gTT**, both having similar swelling characteristics stabilizing at *ca*. 25% from the third scan. As voltage-dependent volumetric capacitances of **PgBT(Ion)2gTT** and **PgBT(TriEG)2gTT** are analogous and higher than that of parent **PgBT(F)2gTT** (Figure S39), the difference in mass uptake between parent/derivatives at anodic potentials can be attributed to less electrolyte uptake by **PgBT(Ion)2gTT** and **PgBT(TriEG)2gTT**.

Second, smaller mass uptakes of *ca*. 2% (above local minima) were observed for **PgBT(Ion)2gTT** and **PgBT(TriEG)2gTT** in regions of reductive applied potential bias (0.2 to −0.4 V), which contrasts with the monotonic mass loss observed for parent **PgBT(F)2gTT** upon its reduction. The mass uptake behavior at cathodic potentials for **PgBT(Ion)2gTT** and **PgBT(TriEG)2gTT** is attributed to the electrostatic attraction of solvated mobile cations into the reduced films,^47,48^ demonstrating cationic mobility within both materials for charge compensation.^37^ The initial reactions of **PgBT(Ion)2gTT** and **PgBT(TriEG)2gTT** films upon increasing the applied potential from −0.4 to 0.8 V in the first scan can be correspondingly analyzed to deduce small amounts of predominantly anion insertion in the beginning, followed by a commensurate level of mostly cation expulsion, preceding the main mass uptake event upon bulk electrochemical oxidation. This implies both cations and anions are active for charge compensation in **PgBT(Ion)2gTT** and **PgBT(TriEG)2gTT**, with cations being already present in their passive-swelled films at open-circuit condition.

Both **PgBT(Ion)2gTT** and **PgBT(TriEG)2gTT** feature cationic expulsion and anionic injection functionality, whereas only counteranions appear to be injected into films of parent **PgBT(F)2gTT** upon its electrochemical oxidation. The introduction of cation expulsion and anionic injection mechanisms into derivatives **PgBT(Ion)2gTT** and **PgBT(TriEG)2gTT** explain their higher volumetric capacitances attained with lower active swelling, when compared with parent **PgBT(F)2gTT**.^37^ Lower maximum and irreversible swelling under electrochemical biases of **PgBT(Ion)2gTT** and **PgBT(TriEG)2gTT** minimize interruption of thin film charge conduction pathways.^18^ Although cationic movements about **PgBT(Ion)2gTT** films can be readily explained to be facilitated by the presence of anionic sulfonate side chains, the mechanism for cation transport in **PgBT(TriEG)2gTT** is less obvious, as stable chelation of Na^+^ cations by single glycol chains has been found to require glycol side chains that are at least tetrameric in length,^49^ and the larger K^+^ cation will be at least as hard to bind. We hypothesize cations in **PgBT(TriEG)2gTT** films are chelated by the adjacent double trimeric ethylene glycol side chains positioned on each benzothiadiazole unit.^49^

The differing EQCM behaviors between parent **PgBT(F)2gTT** and derivatives **PgBT(Ion)2gTT**/**PgBT(TriEG)2gTT** contrast with their equivalent hydrophilicities, as determined through contact angle measurements (Figure S46, Table S2).

**Figure 4:**
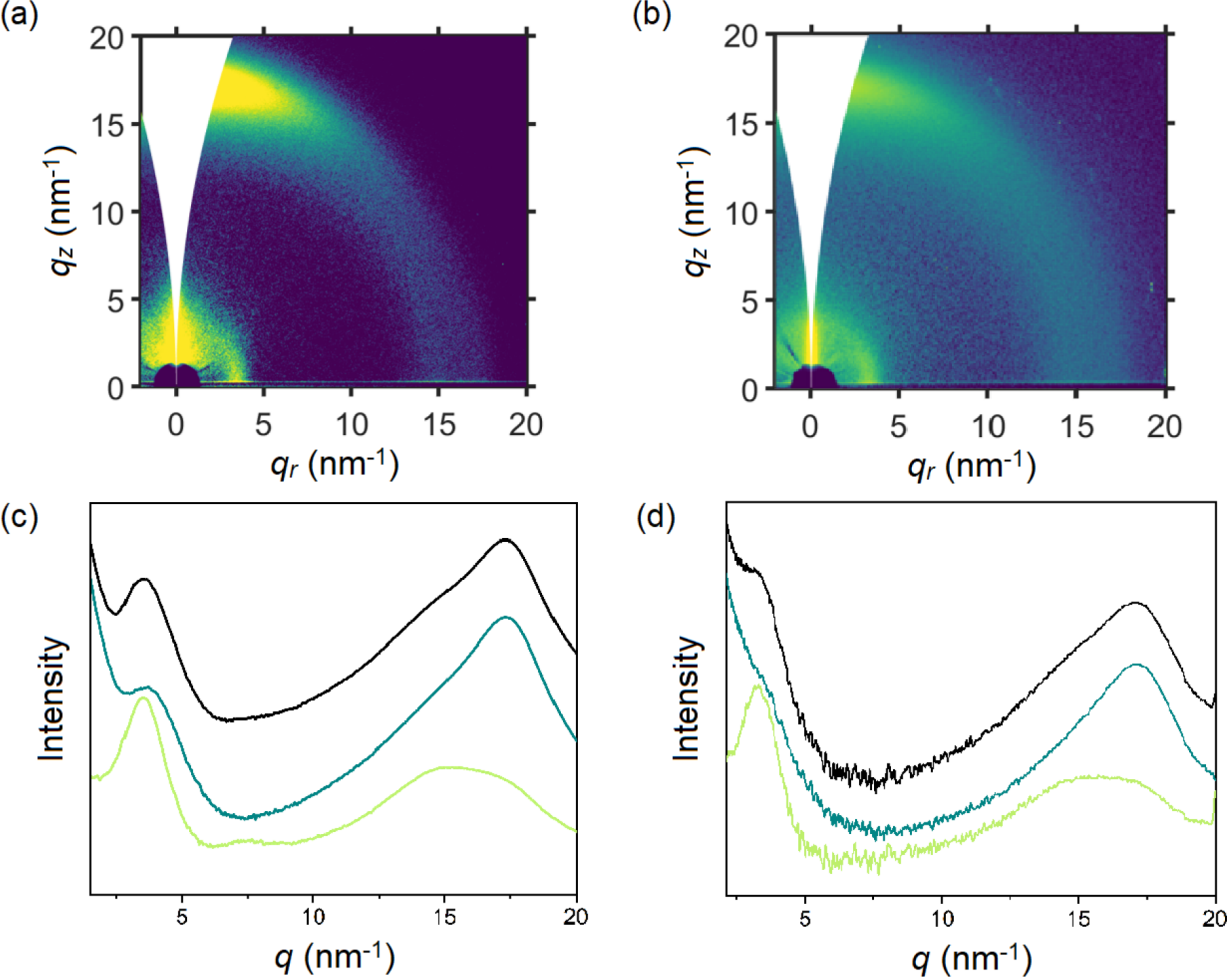
2D GIWAXS patterns of (a) **PgBT(Ion)2gTT** and (b) **PgBT(TriEG)2gTT**, with integrations of scattering shown for (c) **PgBT(Ion)2gTT** and (d) **PgBT(TriEG)2gTT** (isotropic, black; *q*_*z*_, out of plane, dark cyan; *q*_*r*_, in plane, light green).

Thin film morphologies were investigated to decipher differences in charge mobility. Grazing-incidence wide-angle X-ray scattering (GIWAXS, Figure 4) of **PgBT(Ion)2gTT**, **PgBT(TriEG)2gTT**, and the parent **PgBT(F)2gT** all showed (100) “lamellar” peaks at similar *d-spacing* = 1.8 nm (*q* = 3.5 nm^–1^) with π–π stacking peaks at *d-spacing* = 0.36 nm (*q* = 17.3 nm^–1^). Parent **PgBT(F)2gTT** exhibited bimodal crystal orientation,^36^ whereas **PgBT(TriEG)2gTT** packed in a face-on orientation, with the (100) and π–π stacking peaks oriented along the *q*_*r*_ and *q*_*z*_ directions, respectively. Despite stacking mainly face-on to the substrate, **PgBT(Ion)2gTT** films displayed evidence of bimodal crystal orientation, featuring domains oriented in the orthogonal direction, as suggested by the shoulder at *q* = 3.5 nm^–1^ along the *q*_*z*_ axis (Figure 4c).

The bimodal crystal orientation of **PgBT(Ion)2gTT** and its controlled swelling are reasons for its retention of excellent charge transport properties compared to parent **PgBT(F)2gTT**.^36^ For **PgBT(Ion)2gTT**, its high charge mobility is coupled with its improved volumetric capacitance over parent **PgBT(F)2gTT**, facilitated by cationic charge compensation, resulting in its upgraded OECT performance. Despite its similarly controlled swelling properties and high volumetric capacitance, the limited face-on stacking of **PgBT(TriEG)2gTT** may explain its low charge mobility, which hinders overall OECT performance.

## Conclusions

Direct PPM S_N_Ar substitution of the fluorine-functionalized state-of-the-art OECT polymer, **PgBT(F)2gTT**, with anionic side chains has been proven to be a simple and effective means of improving its *p*-type enhancement mode OECT performance. The fully glycolated and hybrid glycol/ionic side chain polymers **PgBT(TriEG)2gTT** and **PgBT(Ion)2gTT**, were successfully derived from **PgBT(F)2gTT**, resulting in higher volumetric capacitances of 147 and 143 F·cm^–3^, respectively (*cf*. 121 F·cm^–3^ for **PgBT(F)2gTT**) with lowered consequential swelling, owing to the addition of cationic charge compensation functionality. However, the limited face-on stacking of **PgBT(TriEG)2gTT** hindered OECT charge transport at 0.26 cm^2^·V^–1^·s^–1^, compromising device performance. With a μC* of 145 cm^–1^·V^–1^·s^–1^, **PgBT(Ion)2gTT** OECTs achieved a significant performance improvement over its parent **PgBT(F)2gTT**, owing to the bimodal crystal orientation (and controlled swelling) of **PgBT(Ion)2gTT** facilitating its excellent charge mobility of 1.02 cm^2^·V^–1^·s^–1^ in devices. These results validate the effectiveness of S_N_Ar anionic side chain attachment by PPM as a convenient and powerful strategy for elevating the volumetric capacitance of glycolated OMIECs without compromising solid-state morphology and charge mobility.

## Methods

### Materials

Synthesis and characterization of polymers, as well as details about commercially obtained materials, are provided in the Supporting Information.

### General Methods

Solution state ^1^H and ^19^F NMR spectra of polymers were collected on a Bruker AVANCE 400 or 500 MHz spectrometer at an elevated temperature of 328 K, with internal referencing for chemical shifts (δ) using the solvent residual signal. Solid state total reflectance ATR-IR spectra were obtained on an Agilent CARY 630 FTIR spectrometer. Matrix-assisted laser desorption/ionization time-of-flight (MALDI-ToF) mass spectrometry data were collected on a Micromass MALDI-ToF analyzer, with collections operated in positive mode along mass ranges (*m*/*z*) tuned according to the sample. Polymers were cast in a 2,2′:5′,2″-terthiophene matrix from THF solutions, with the addition of benzenesulfinic acid sodium salt to aid the ionization of PgBT(TriEG)2gTT. The *M*_n_, *M*_w_, and *Đ* (against polystyrene standards) of polymers dissolved in HPLC-grade DMF containing 0.1 M LiCl were determined using an Agilent 1260 Infinity GPC instrument fitted with a guard column and two PLgel 10 μm mixed-B 7.5 × 300 mm columns, running at an oven temperature of 60 °C and flow rate of 1.0 mL/min. Differential scanning calorimetry (DSC) traces of polymers between 40 and 300 °C were collected on a Mettler DSC822e differential scanning calorimeter, at a heating rate of 10 °C/min under a N_2_ environment. UV/vis data were collected using an Agilent CARY 60 UV/vis spectrometer interfaced with SCAN software. CHCl_3_ was used for solution state measurements of polymers, whereas solid state measurements were collected using thin films drop-cast onto precleaned FTO slides. Drop-casting of thin films were conducted using 2.5 mg/mL polymer solutions in CHCl_3_. An Agilent Eclipse Fluorescence spectrophotometer was used for the collection of fluorescence data of polymers dissolved in CHCl_3_. GIWAXS patterns were collected at the ALBA synchrotron in Spain, on polymer thin films spun onto Si wafer substrates by dynamic spin-coating of 10 mg/mL solutions in CHCl_3_ at 2000 rpm. Thicknesses of films were measured by a Dektak profilometer. The pendant drop method (First Ten Angstroms FTA1000B) was applied to determine the contact angle of deionized water on polymer thin films on glass, formed by dynamic spin-coating at 2000 rpm using 10 mg/mL solutions in CHCl_3_.

### Electrochemistry

Solid state cyclic voltammetry (CV) and square wave voltammetry (SQW) measurements of polymer thin films were conducted with a Metrohm Autolab PGSTAT101 Electrochemical Analyzer interfaced to NOVA software. A custom-made one compartment three electrode electrochemical cell was used for all measurements, featuring a 2 cm^2^ FTO-on-glass working electrode (WE) and a Pt mesh counter electrode. An Ag/AgCl aqueous reference electrode was used for measurements in 0.1 M KCl/H_2_O. An Ag/Ag^+^ nonaqueous reference electrode was applied for measurements in 0.1 M [*n*-Bu_4_N]PF_6_/MeCN, with the addition of a ferrocene internal reference. Saturation of electrolyte with N_2_ by bubbling for 20 min was performed to deoxygenate before measurements were taken. Thin films of polymers were drop-cast onto the conductive side of precleaned FTO slides from 2.5 mg/mL solutions of polymer in CHCl_3_. Conversion to energy levels was conducted by assuming the Ag/AgCl reference occurs at −4.6 eV.

### UV/Vis Spectroelectrochemistry (SEC)

UV/vis SEC of the polymer thin films in 0.1 M KCl/H_2_O electrolyte were conducted using a sample holder from redox.me (MM SPECTRO-EFC), with the recording of spectroscopic data using Ocean Optics UV/vis (FLAME-S) and NIR (NQ-512) spectrometers, paired with an Ocean Optics Halogen light source (HL-2000-HFSA), controlled using OceanView software. The SEC cell featured a Pt mesh counter electrode, an Ag/AgCl aqueous reference electrode, and a 2 cm^2^ FTO-on-glass WE, onto which polymers were coated by dynamic spin-coating at 2000 rpm using 10 mg/mL solutions. The applied potential was controlled incrementally using a Metrohm μStat-i 400s Impedance Analyzer interfaced to DropSens software. Equilibrated spectra were recorded for various applied potentials.

### OECT Device Fabrication and Characterization

For the fabrication of OECTs (Scheme S1), gold (Au, 45 nm) and chromium (Cr, 5 nm) were first applied as the source and drain electrodes, which were patterned on the glass substrate by conventional lift-off photolithography. To prepare the OECT active layers, polymer solutions in CHCl_3_ at 10 mg/mL were spin-coated onto the substrates at 2000 rpm for 60 s. CYTOP (CTL-809M, Asahi Glass Co.) was then spin-coated at 2000 rpm and then baked at 100 °C for 1 h for active layer patterning. To improve the hydrophilicity of the CYTOP surface, it was treated with O_2_ plasma for 10 s, upon which the positive photoresist (GXR-601, Microchemicals GmbH) was spin-coated on and patterned for the channel area (channel length: 20, 40, 60, 80 μm, width: 80 μm) through a conventional photolithography process. Next, other areas of the device to be exposed to the electrolyte, except for the channel part, were passivated with an epoxy-based photoresist (SU-8 2002, MicroChem Co.). Lastly, the device was immersed in a bath with fluorinated solvent (HFE-7300, 3M) and stirred at 50 °C overnight to dissolve the CYTOP layer.

The performances of OECTs were measured using two Keithley 2400 source meters, applying custom MATLAB code. An Ag/AgCl electrode and 100 mM NaCl were employed as the gate electrode and OECT electrolyte, respectively.

### Computational Details

All computational modeling was carried out in Gaussian 09 using density functional theory (DFT) applied at the B3LYP/6-31G(d,p) level, with use of GaussView 5.0 to perform structural and orbital visualizations. Geometry optimization and natural bond orbital population analyses were carried out on trimeric units of the conjugated backbone common to both **PgBT(Ion)2gTT** and **PgBT(TriEG)2gTT**. Side chain positions along the polymer backbone were replaced by methoxy groups for computational feasibility.

### Electrochemical Quartz Crystal Microbalance (EQCM)

Electrogravimetric measurements were performed on a QCM200 quartz crystal microbalance (Stanford Research System, SRS) using 5 MHz AT-cut gold-coated quartz crystals as substrates. The substrates were cleaned by successive ultrasonication in acetone, deionized water, and propan-2-ol for 10 min each. The absolute frequencies of each substrate (*F*_0_) were first recorded on the QCM. Polymers were then dynamic spin-coated onto the substrates at 2000 rpm for 60 s, from 10 mg/mL polymer solutions in CHCl_3_, and any excess material outside of the gold-coated substrate area was meticulously wiped off using acetone wetted swabs. These polymer-coated crystals were placed on the QCM holder to measure their absolute frequencies (*F*_1_), from which the frequency changes due to the load of films (Δ*f*_1_) can be calculated using Δ*f*_1_ = *F*_1_ – *F*_0_.

To monitor the mass change of the films during electrochemical processes, the QCM holder was immersed in a three-electrode electrochemical cell with argon-saturated 0.1 M KCl/H_2_O electrolyte, within which the polymer-coated crystal serves as the WE, alongside a Pt wire counter electrode, and an Ag/AgCl aqueous reference electrode. A Metrohm Autolab PGSTAT302N interfaced to NOVA software was used to control the applied potential and simultaneously record the external potential change (Δ*V*) fed by the QCM. The QCM monitored the frequency change of the polymer films (Δ*f*_2_) during electrochemical biasing and converted it into Δ*V* through a preset scaling factor (of 200 Hz/V). The change in frequency (Δ*f*_1_ and Δ*f*_2_) can be converted to the change in mass using the Sauerbrey equation, assuming a rigid film (*m*_*dryfilm*_ = mass of dry pristine film, Δ*m* = mass change during an electrochemical process). Here, we normalized Δ*m* using
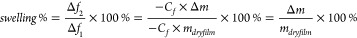
where *C*_*f*_ is the sensitivity factor for the quartz crystal substrate used.

## Supporting Information Available

Material synthesis and characterization details (NMR spectra, MALDI traces, IR spectra, DSC traces, GPC traces, and UV/vis spectra), DFT calculation results, electrochemistry, UV/vis SEC, OECT plots as well as contact angle photographs (PDF)
