# Supplementary material for: Enhanced Organic Electrochemical Transistor Performance of Donor–Acceptor Conjugated Polymers Modified with Hybrid Glycol/Ionic Side Chains by Postpolymerization Modification
Source: Chem Mater. 2023 Apr 11;35(8):3290–9. doi: 10.1021/acs.chemmater.3c00327 (PMC10134426; doi:10.1021/acs.chemmater.3c00327)
Supplement: Supplementary file 1 — cm3c00327_si_001.pdf [file cm3c00327_si_001.pdf]

Supplementary Information

Enhanced Organic Electrochemical Transistor Performance of Donor-Acceptor Conjugated Polymers Modified with Hybrid Glycol/Ionic Sidechains by Post-Polymerization Modification

Bowen Ding,[a†] Il-Young Jo,[b†] Hang Yu,[c] Ji Hwan Kim,[b] Adam V. Marsh,[d] Edgar Gutiérrez-Fernández,[e] Nicolás Ramos,[e] Charlotte L. Rapley,[a] Martina Rimmele,[a] Qiao He,[a] Jaime Martín,[e,f] Nicola Gasparini,[a] Jenny Nelson,[c] Myung-Han Yoon[b]* and Martin Heeney[a,d]*

[a]Department of Chemistry and Centre for Processable Electronics, Imperial College London, Molecular Sciences Research Hub (White City Campus), 80 Wood Lane Shepherd's Bush, London W12 0BZ, United Kingdom

[b]School of Materials Science and Engineering, Gwangju Institute of Science and Technology, 123 Cheomdangwagi-ro, Buk-gu, Gwangju 61005, Republic of Korea

[c]Department of Physics and Centre for Processable Electronics, Imperial College London, South Kensington Campus, London SW7 2AZ, United Kingdom

[d]KAUST Solar Center, Physical Sciences and Engineering Division (PSE), King Abdullah University of Science and Technology (KAUST), Thuwal, 23955-6900, Saudi Arabia

[e]POLYMAT and Polymer Science and Technology Department, Faculty of Chemistry, University of the Basque Country UPV/EHU, Manuel de Lardizabal 3, 20018 Donostia – San Sebastián, Spain

[f]Universidade da Coruña, Grupo de Polímeros, Departamento de Física e Ciencias da Terra, Centro de Investigacións Tecnolóxicas (CIT), Esteiro, 15471 Ferrol, Spain

†These authors contributed equally.

*Corresponding Authors: martin.heeney@kaust.edu.sa and mhyoon@gist.ac.kr

EXPERIMENTAL DETAILS

Materials

All reagents and solvents were obtained commercially and used without further purification unless otherwise stated. KCl and [*n*-Bu_4N]PF_6 for electrochemistry were recrystallized twice in H_2O and EtOH respectively. All substrates (including 2 cm^2 fluorine doped tin oxide (FTO) slides for electrochemistry and spectroelectrochemistry, glass slides for general thin film analyses, glass substrates from Eagle XG, Corning Inc. for organic electrochemical transistor (OECT) fabrication, Si wafer pieces for grazing-incidence wide-angle X-ray scattering (GIWAXS)) were cleaned thoroughly by sonication before use; first in soapy water, then thrice each in distilled water, acetone and finally propan-2-ol, and subsequently dried under a stream of N_2. Si wafer substrates for GIWAXS were further treated with a UV-ozone cleaner prior to spin-coating. **PgBT(F)2gTT** used herein was from the same batch that was synthesized for a previous study.[1] Deuterated $CDCl_3$ for NMR was obtained from Fluorochem.

Synthesis

PgBT(Ion)2gTT

PgBT(F)2gTT (203 mg, 0.215 mmol), sodium 3-hydroxypropane-1-sulfonate (69.3 mg, 0.427 mmol) and NaOtBu (40.7 mg, 0.423 mmol) were combined in a dried 20 mL microwave vial, which was sealed and purged under N_2 for 20 mins. Then, dry DMF (5 mL) was added to the reaction, and the resulting mixture was deoxygenated by N_2 bubbling for another 20 mins, before being heated in a covered oil bath at 120 °C for 3 days. Upon completion, the reaction was cooled to RT and precipitated into MeOH (200 mL). The resulting suspension was sonicated for 15 mins and filtered into a Soxhlet thimble. The solids were washed by Soxhlet extraction with MeOH, after which the polymer was extracted with $CHCl_3$. The dark blue $CHCl_3$ fraction was concentrated to <5 mL by rotary evaporation and re-precipitated into MeOH (100 mL). The suspended solids were isolated by filtration to give the title polymer as a blue-black solid (184 mg, 79%). GPC (DMF, 333 K): M_n = 19 KDa, M_w = 24 KDa and Đ = 1.3 (against polystyrene). ^{1}H NMR ($CDCl_3$, 500 MHz, 328 K): δ 8.60 – 8.00 (br, 4H), 4.90 – 3.05 (br m, 51H) ppm. ^{19}F NMR ($CDCl_3$, 471 MHz, 328 K): no signal.

PgBT(TriEG)2gTT

PgBT(F)2gTT (134 mg, 0.142 mmol), NaOtBu (27.4 mg, 0.285 mmol) and triethyleneglycol monomethylether (45.6 µL, 46.8 mg, 0.285 mmol) and were combined in a dried 20 mL microwave vial, which was sealed and purged under N_2 for 20 mins. Then, dry DMF (6.5 mL) was added to the reaction, and the resulting mixture was deoxygenated by N_2 bubbling for another 20 mins, before being placed in a covered oil bath at 120 °C for 1 day. Upon completion, the

reaction was cooled to RT and precipitated into MeOH (100 mL). The resulting suspension was sonicated for 15 mins and filtered into a Soxhlet thimble. The solids were washed by Soxhlet extraction with MeOH, after which the polymer was extracted with $CHCl_3$. The dark blue $CHCl_3$ fraction was concentrated to <5 mL by rotary evaporation and re-precipitated into MeOH (100 mL). The suspended solids were isolated by filtration to give the title polymer as a blue-black solid (134 mg, 87%). GPC ($CHCl_3$, 333 K): M_n = 21 KDa, M_w = 34 KDa and Đ = 1.6 (against polystyrene). ^{1}H NMR ($CDCl_3$, 400 MHz, 328 K): δ 9.06 (br, 1H), 8.47 (br, 1H), 8.33 (br, 1H), 7.49 (br, 1H), 4.57 (br m, 8H), 4.43 (br m, 8H), 4.33 – 3.45 (br m, 32H), 3.35 (br m, 12H, OCH_3) ppm. ^{19}F NMR ($CDCl_3$, 377 MHz, 328 K): no signal.

TABLES AND FIGURES

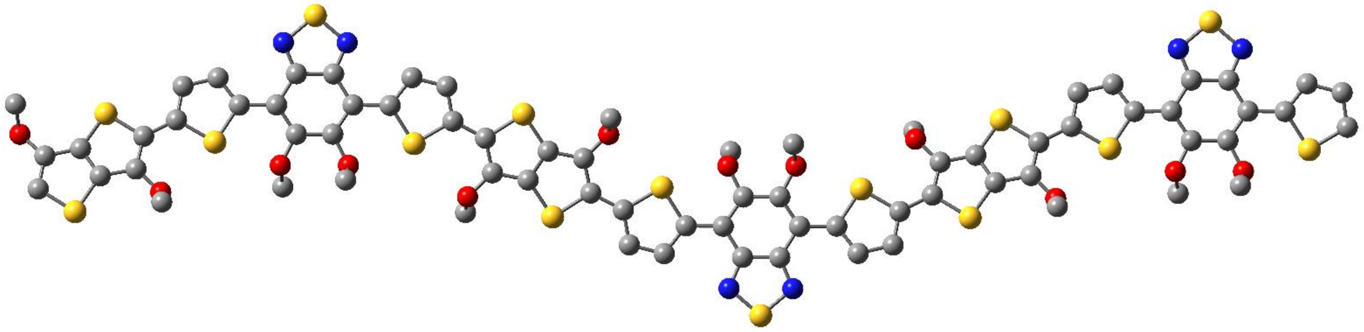

Figure S1: DFT optimized trimeric model of the conjugated backbone common to both **PgBT(Ion)2gTT** and **PgBT(TriEG)2gTT** ($E_{g,DFT}$ = 1.94 eV) performed at the B3LYP/6-31G(d,p) level of theory. Hydrogens omitted for clarity. Atom colours are; carbon – grey, oxygen – red, nitrogen – blue and sulfur – yellow.

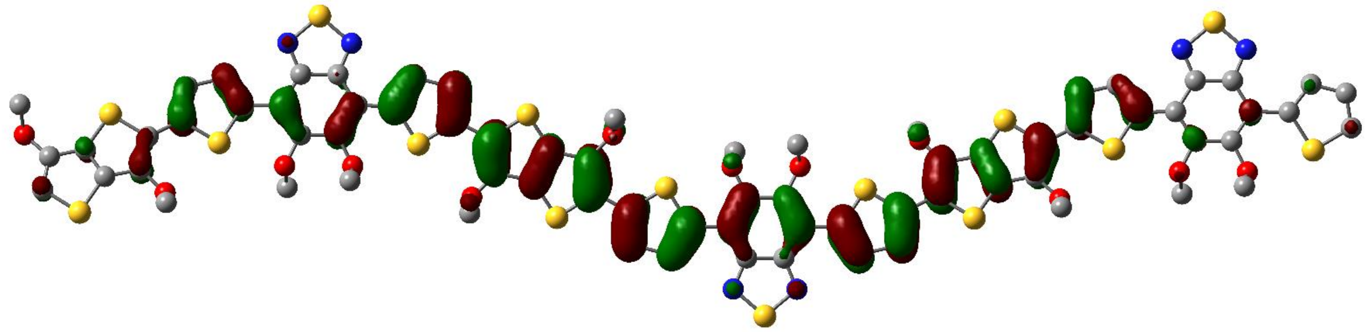

Figure S2: DFT optimized trimeric model of the conjugated backbone common to both **PgBT(Ion)2gTT** and **PgBT(TriEG)2gTT**, performed at the B3LYP/6-31G(d,p) level of theory. HOMO distribution along backbone shown (HOMO at -4.57 eV). Hydrogens omitted for clarity. Atom colours are; carbon – grey, oxygen – red, nitrogen – blue and sulfur – yellow.

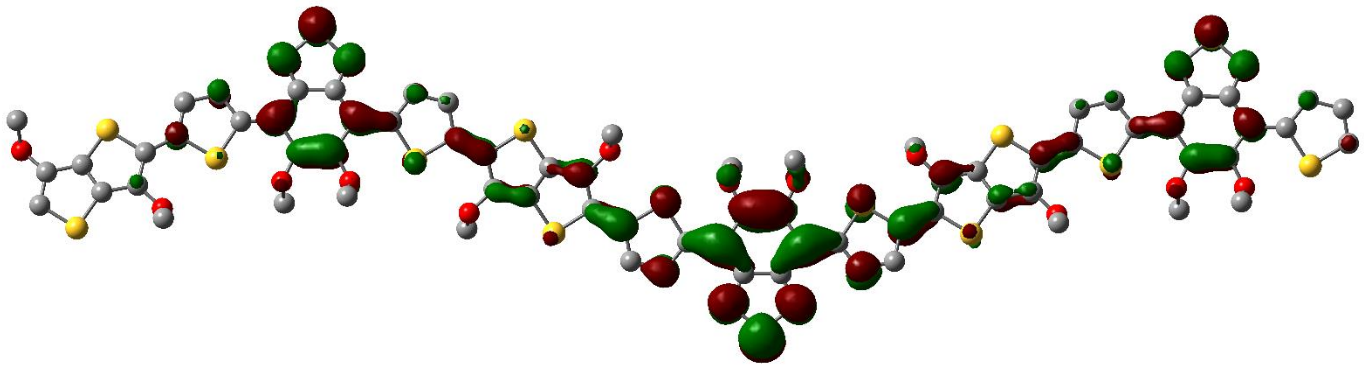

Figure S3: DFT optimized trimeric model of the conjugated backbone common to both **PgBT(Ion)2gTT** and **PgBT(TriEG)2gTT**, performed at the B3LYP/6-31G(d,p) level of theory. LUMO distribution along backbone shown (LUMO at -2.63 eV). Hydrogens omitted for clarity. Atom colours are; carbon – grey, oxygen – red, nitrogen – blue and sulfur – yellow.

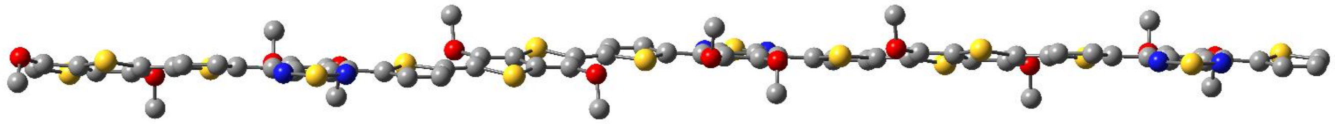

Figure S4: DFT optimized trimeric model of the conjugated backbone common to both **PgBT(Ion)2gTT** and **PgBT(TriEG)2gTT**, performed at the B3LYP/6-31G(d,p) level of theory. Planarity of backbone shown. Hydrogens omitted for clarity. Atom colours are; carbon – grey, oxygen – red, nitrogen – blue and sulfur – yellow.

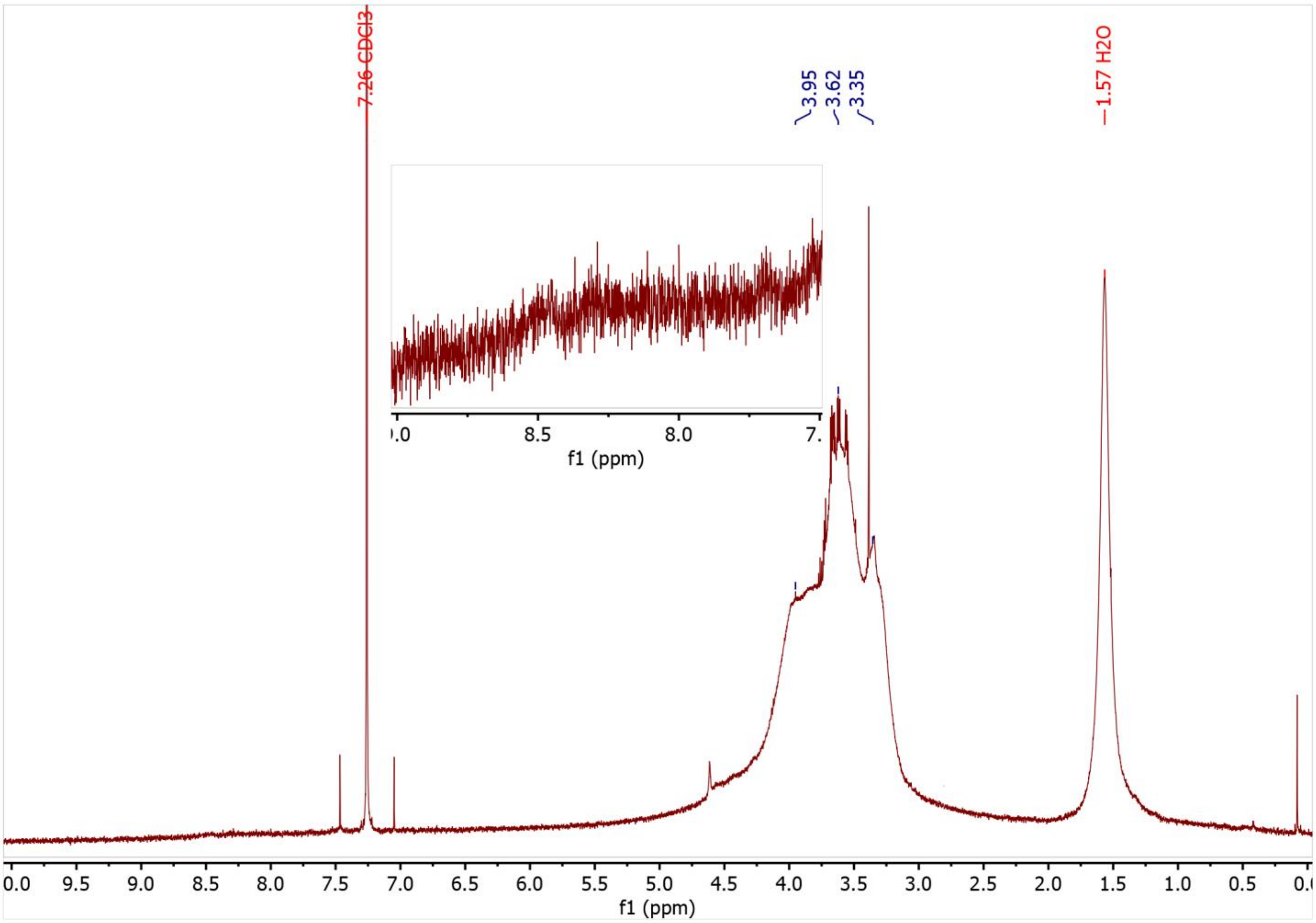

Figure S5: ^{1}H NMR of **PgBT(Ion)2gTT** in $CDCl_3$ at 328 K.

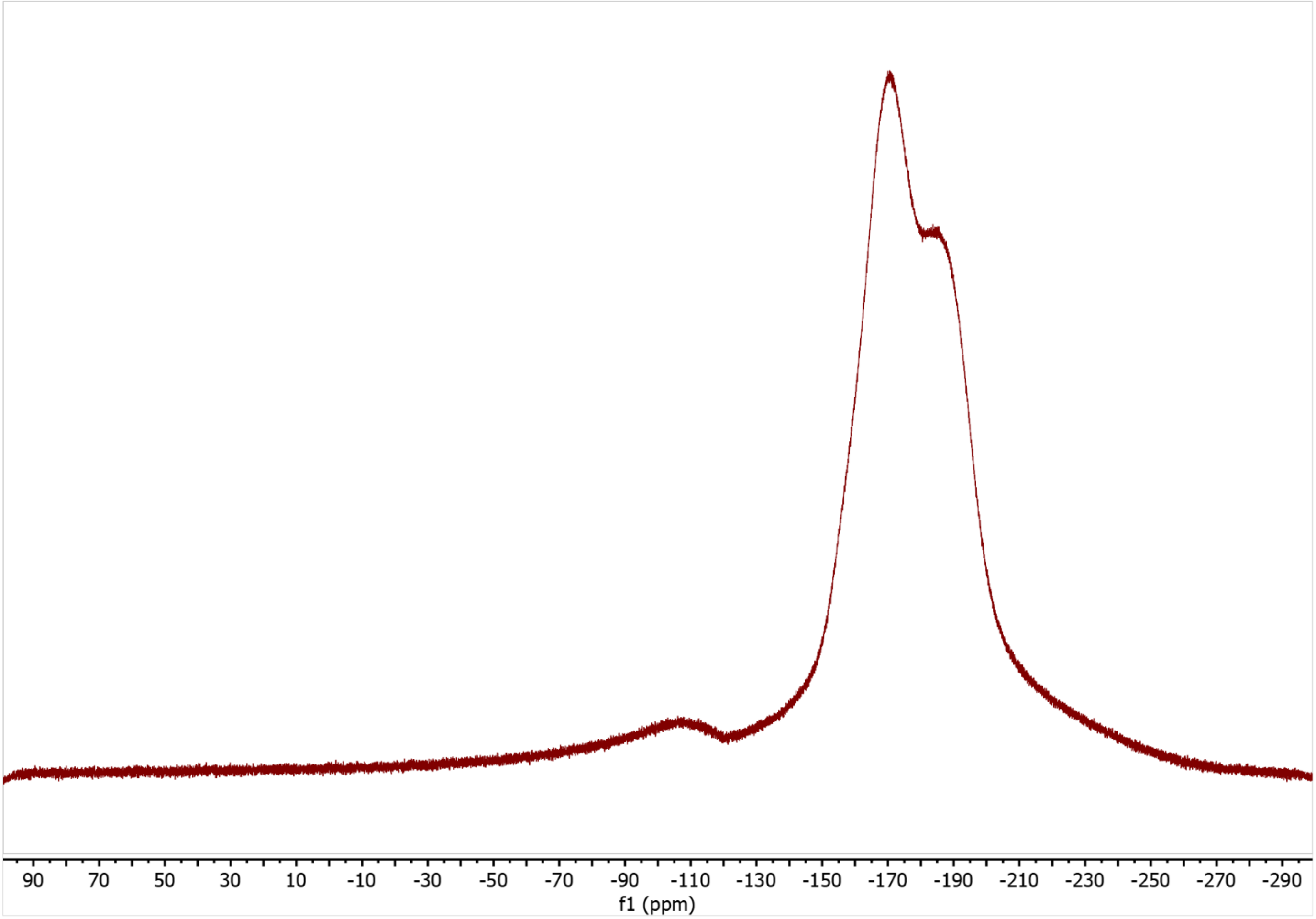

Figure S6: ^{19}F NMR of **PgBT(Ion)2gTT** in $CDCl_3$ at 328 K.

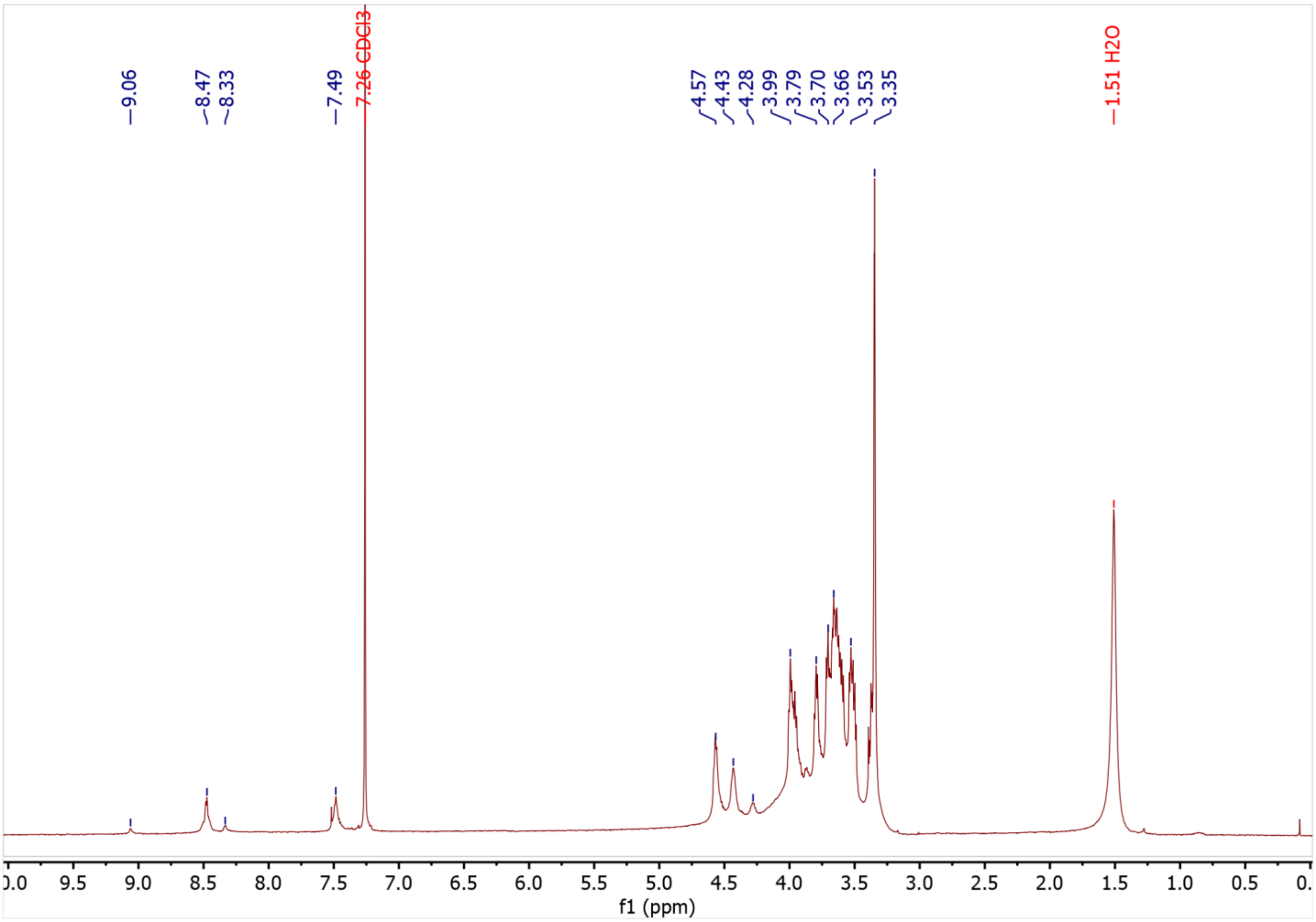

Figure S7: ^{1}H NMR of **PgBT(TriEG)2gTT** in $CDCl_3$ at 328 K.

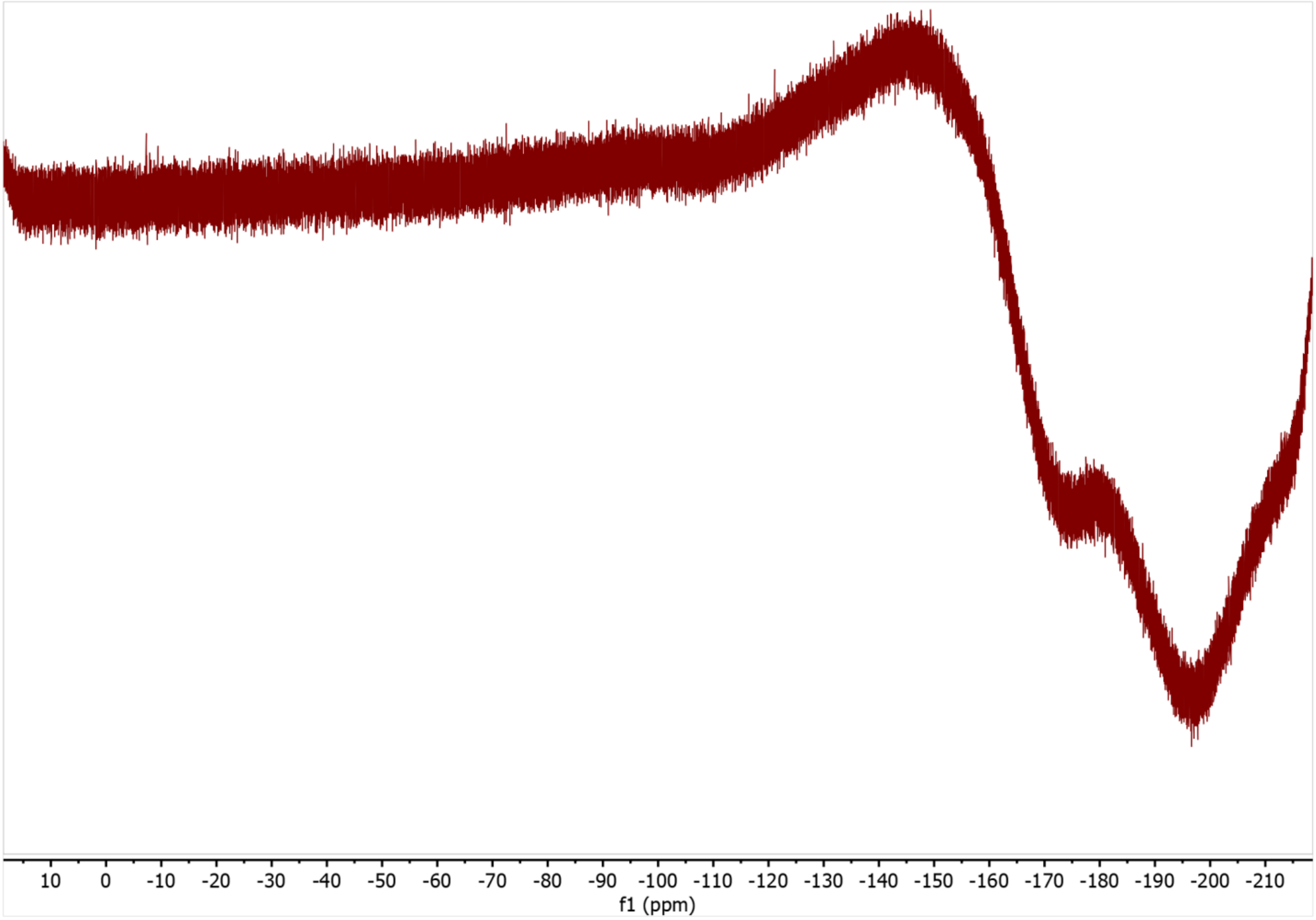

Figure S8: ^{19}F NMR of **PgBT(TriEG)2gTT** in $CDCl_3$ at 328 K.

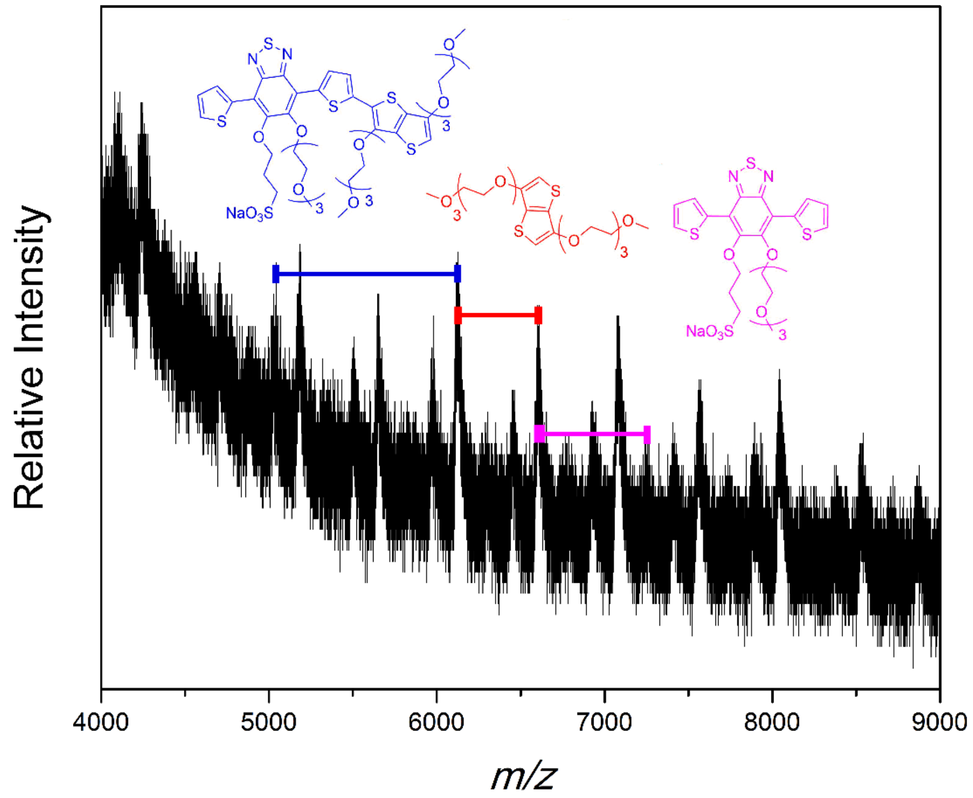

Figure S9: MALDI-ToF of **PgBT(Ion)2gTT**.

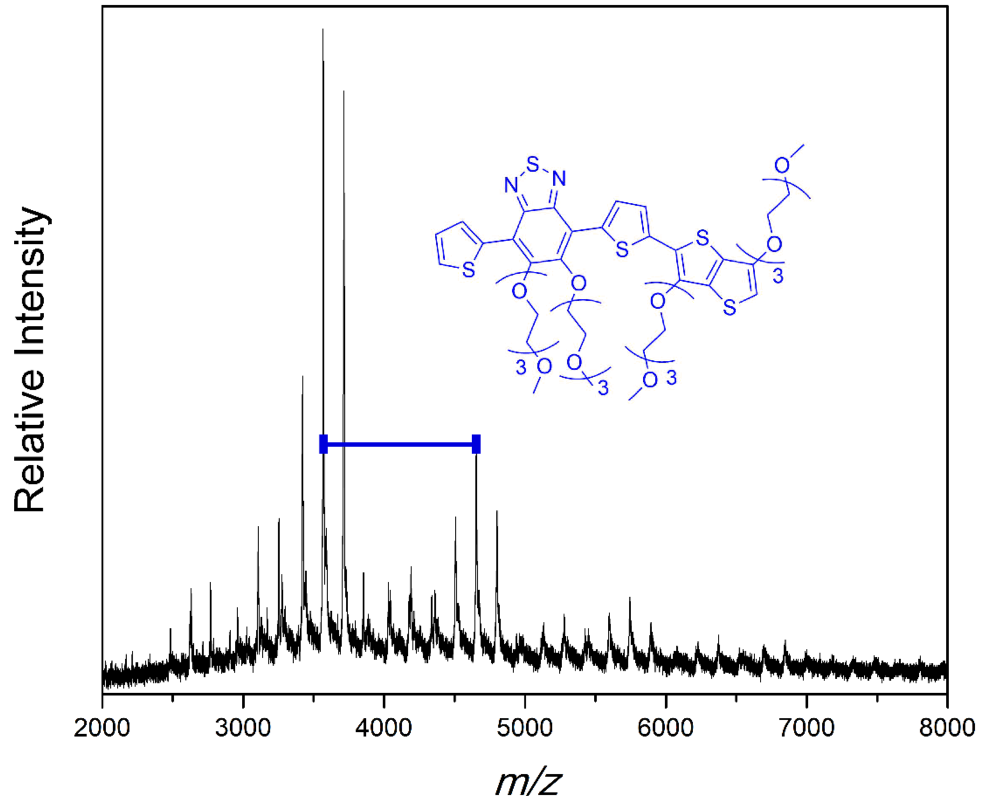

Figure S10: MALDI-ToF of **PgBT(TriEG)2gTT**.

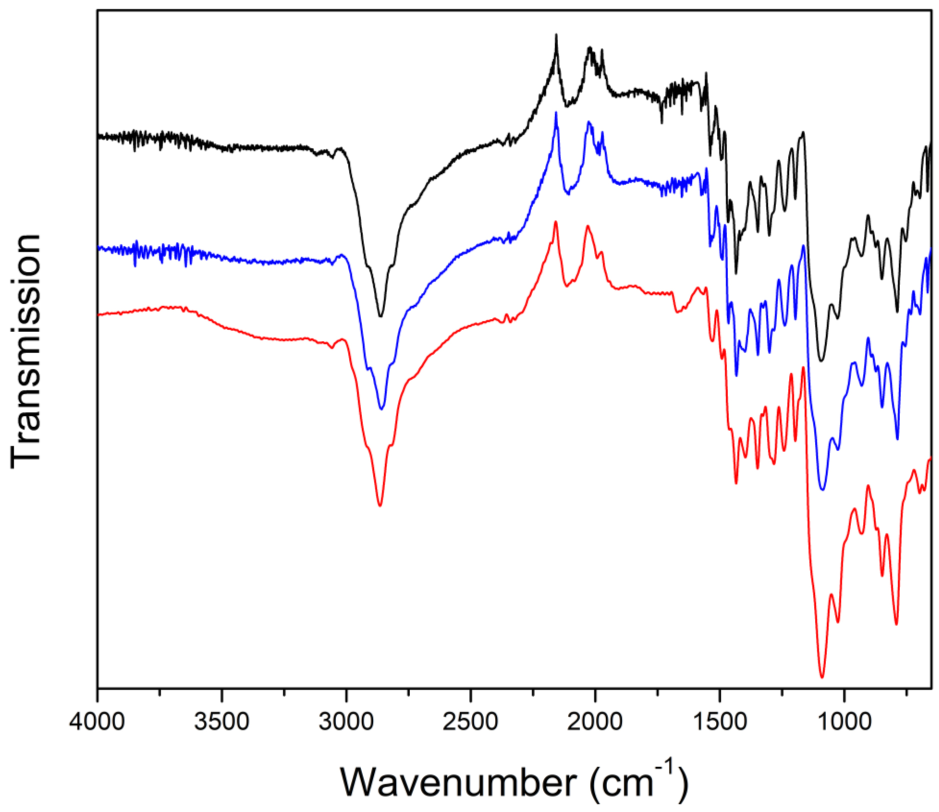

Figure S11: ATR-IR spectra of **PgBT(F)2gTT** (black), **PgBT(Ion)2gTT** (blue) and **PgBT(TriEG)2gTT** (red).

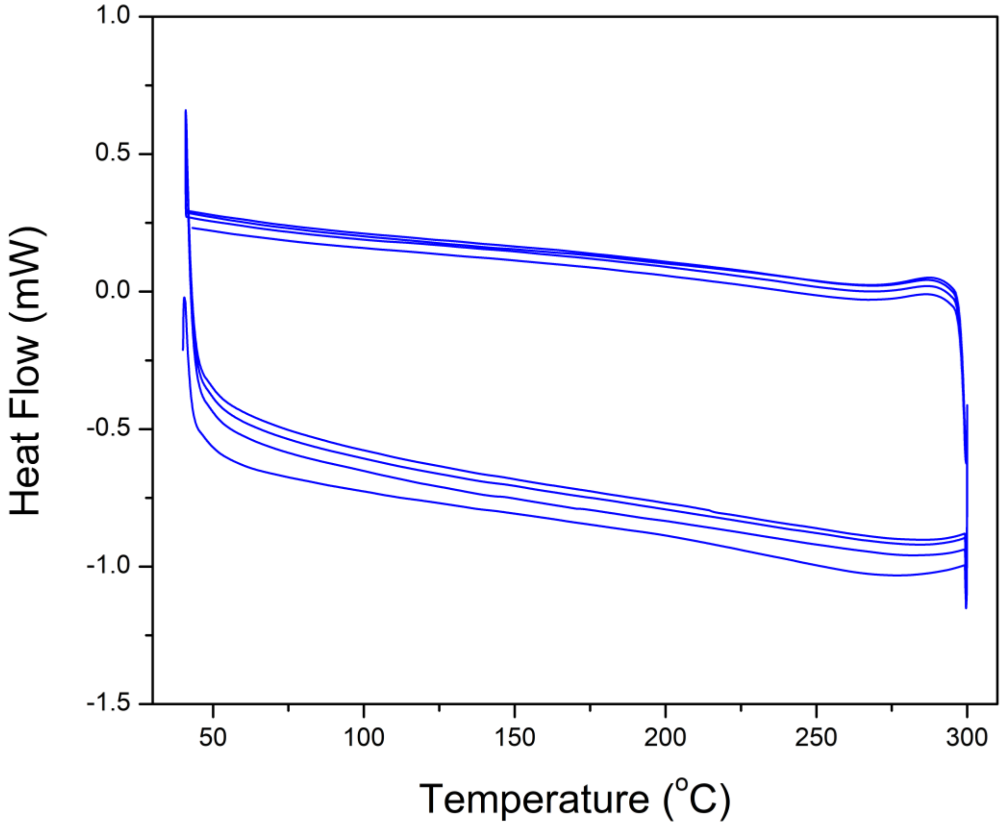

Figure S12: DSC trace (3 cycles) of **PgBT(Ion)2gTT** between 40 – 300 °C.

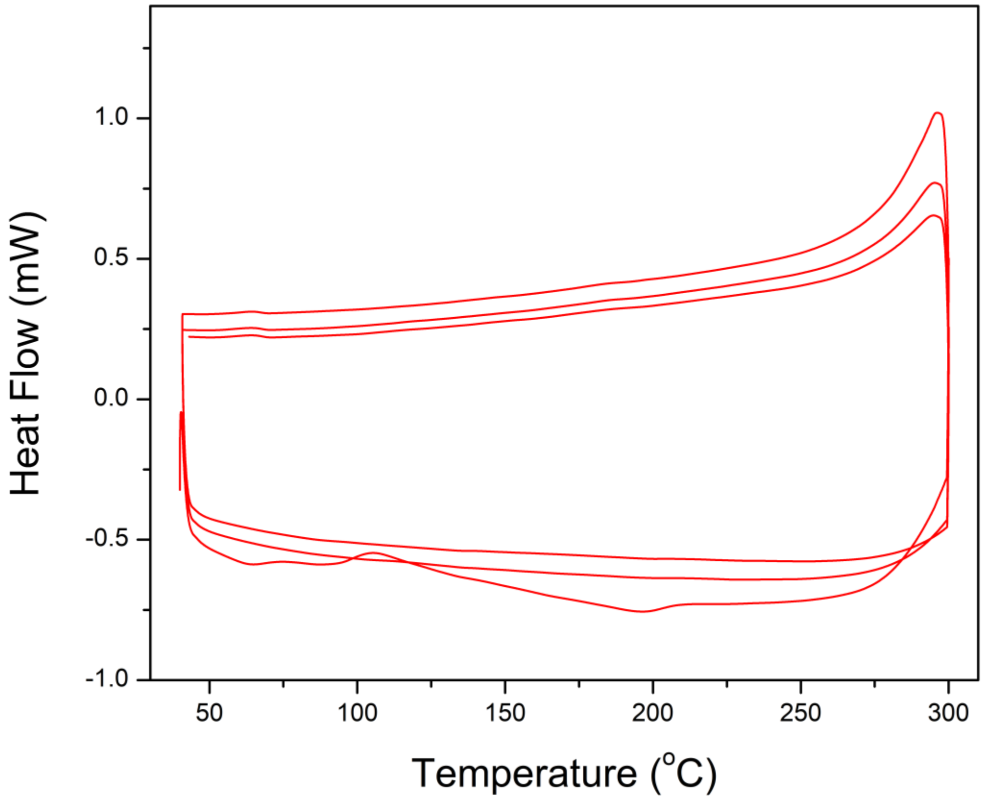

Figure S13: DSC trace (3 cycles) of **PgBT(TriEG)2gTT** between 40 – 300 °C.

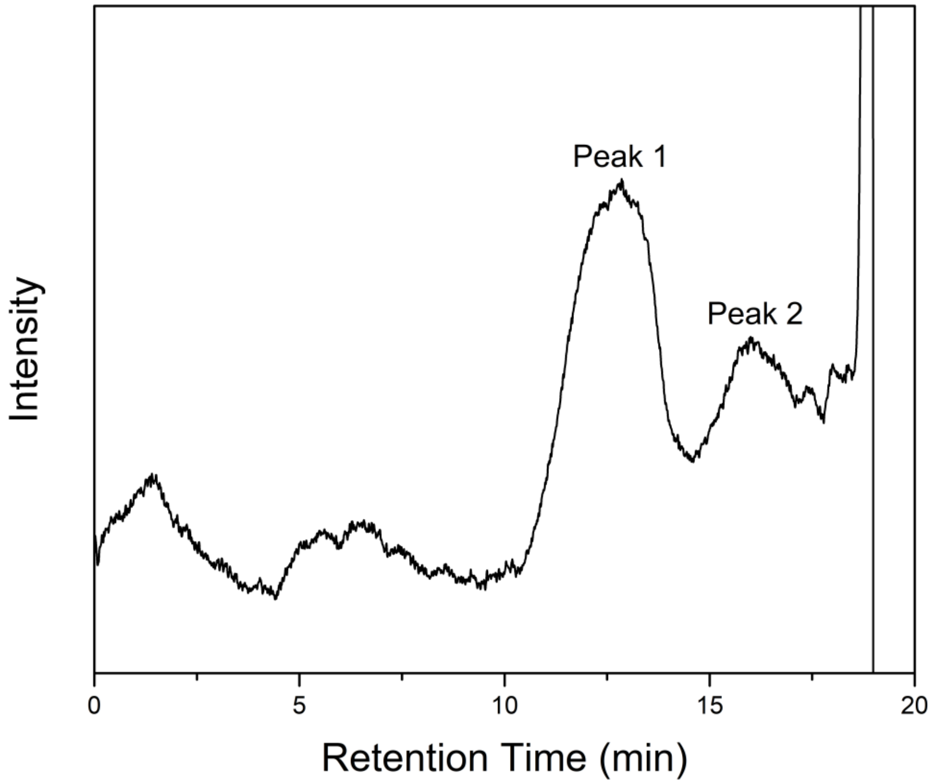

Figure S14: GPC trace of **PgBT(Ion)2gTT** in DMF at 60 °C. Note the bimodal elution peak attributed to aggregation (peak labelled "Peak 2" integrated to estimate M_n, M_w and Đ, against polystyrene standards).

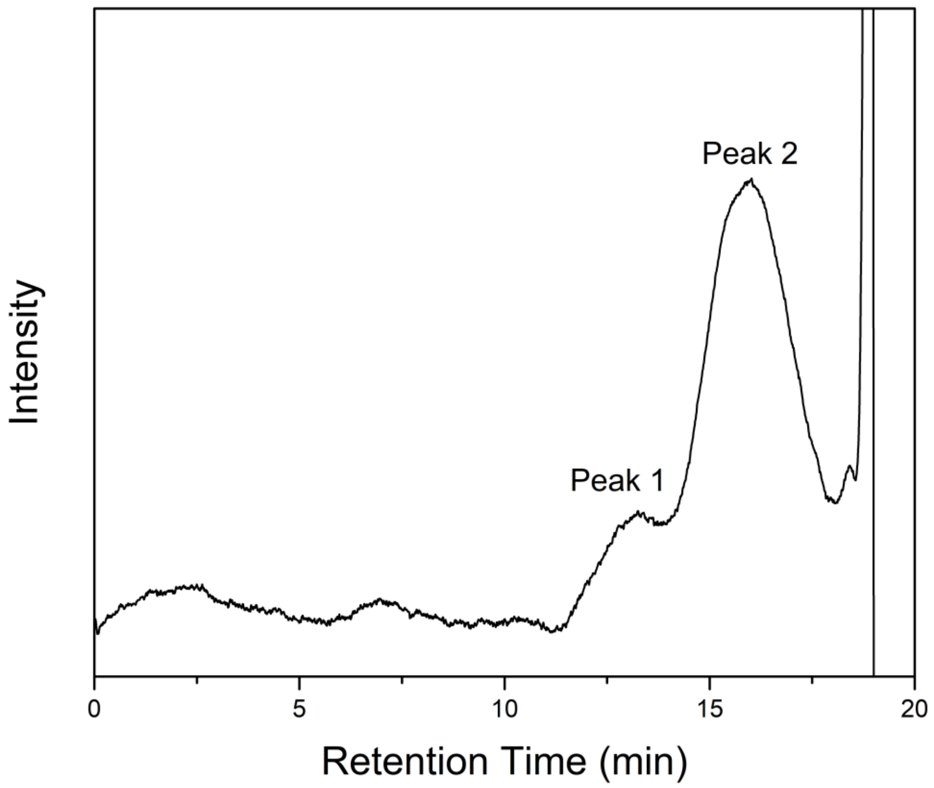

Figure S15: GPC trace of **PgBT(TriEG)2gTT** in DMF at 60 °C. Note the bimodal elution peak attributed to aggregation (peak labelled "Peak 2" integrated to estimate M_n, M_w and Đ against polystyrene standards).

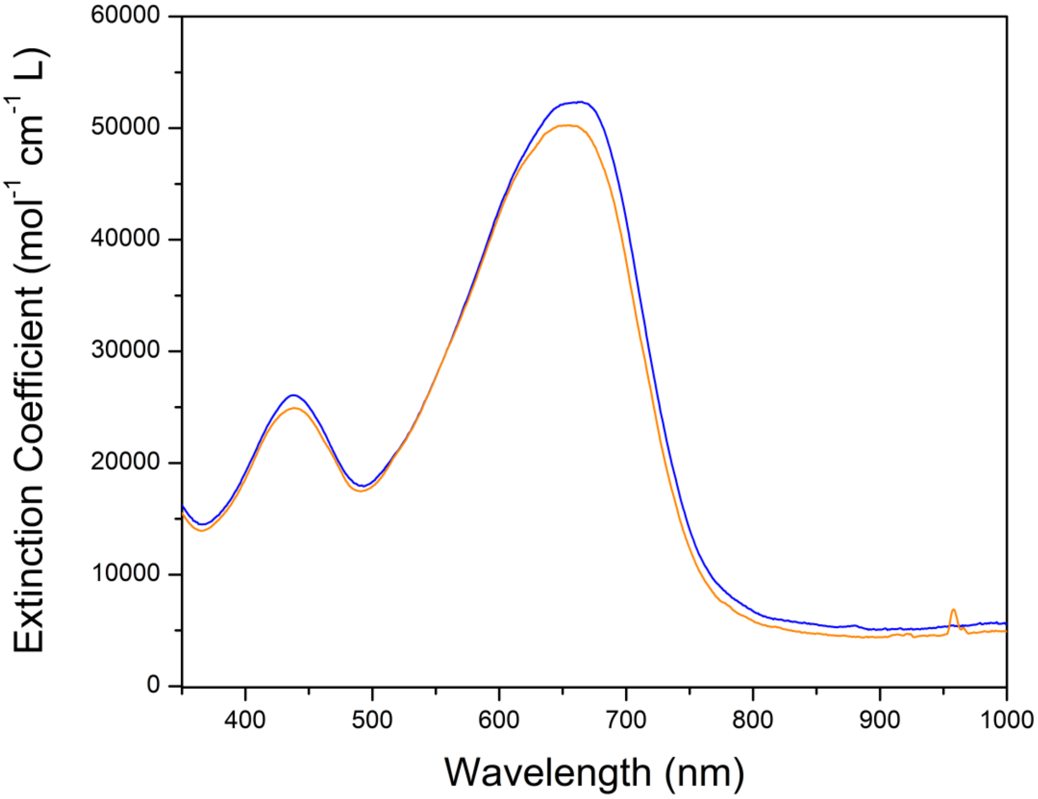

Figure S16: Solution state UV/Vis sectra of **PgBT(Ion)2gTT** in $CHCl_3$, at RT (blue) and upon heating to *ca.* 50°C (orange).

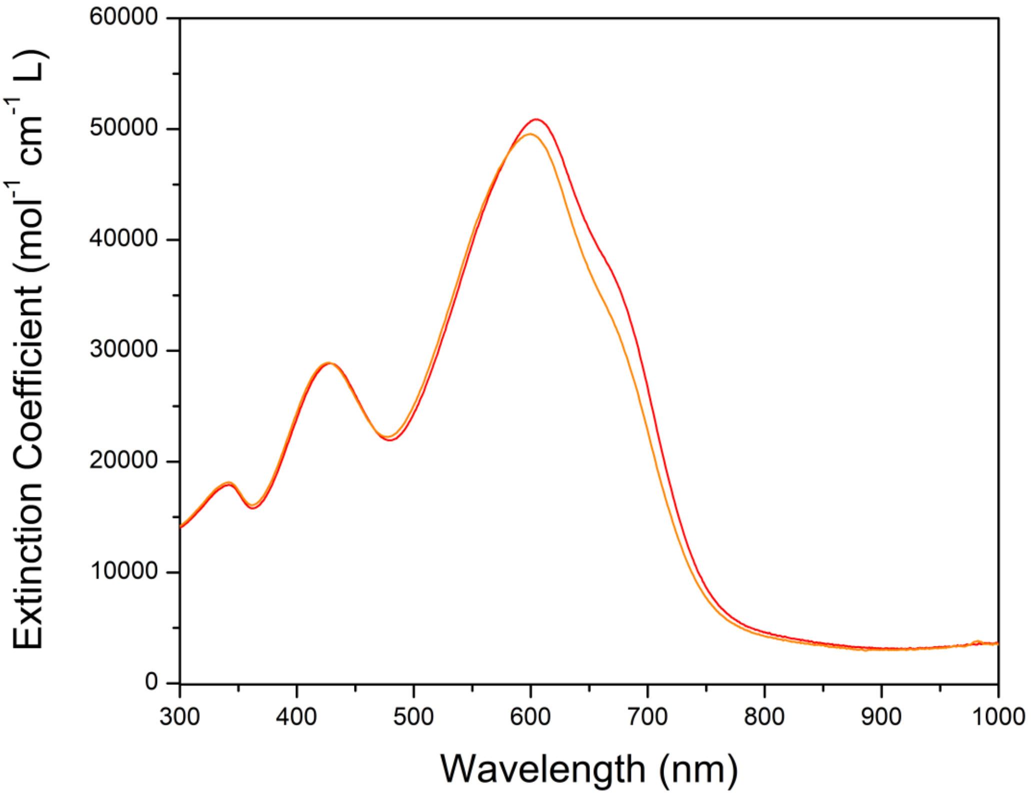

Figure S17: Solution state UV/Vis spectra of **PgBT(TriEG)2gTT** in $CHCl_3$, at RT (red) and upon heating to *ca*. 50°C (orange).

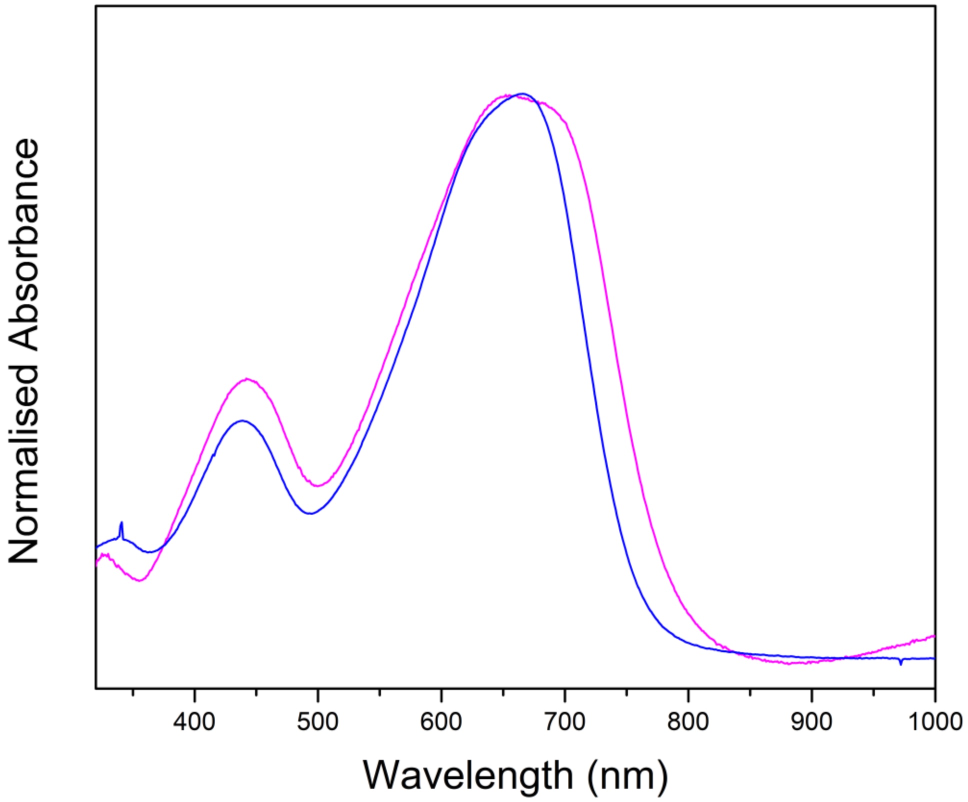

Figure S18: Solid state thin film (magenta) and $CHCl_3$ solution state (blue) UV/Vis spectra of **PgBT(Ion)2gTT**.

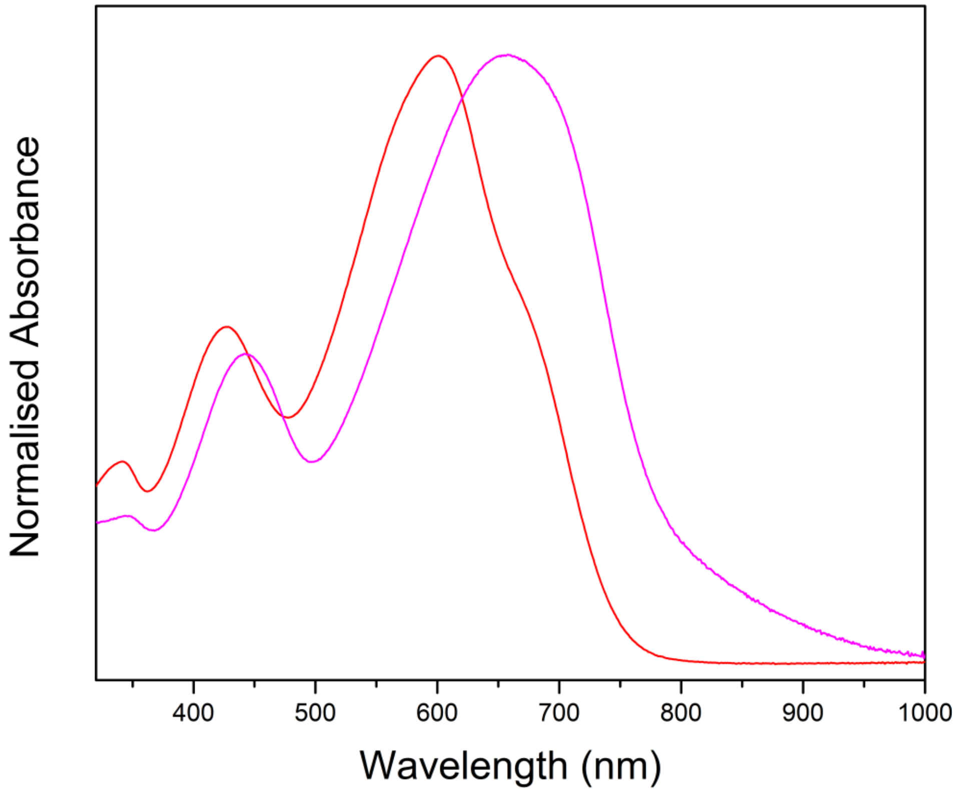

Figure S19: Solid state thin film (magenta) and $CHCl_3$ solution state (red) UV/Vis spectra of **PgBT(TriEG)2gTT**.

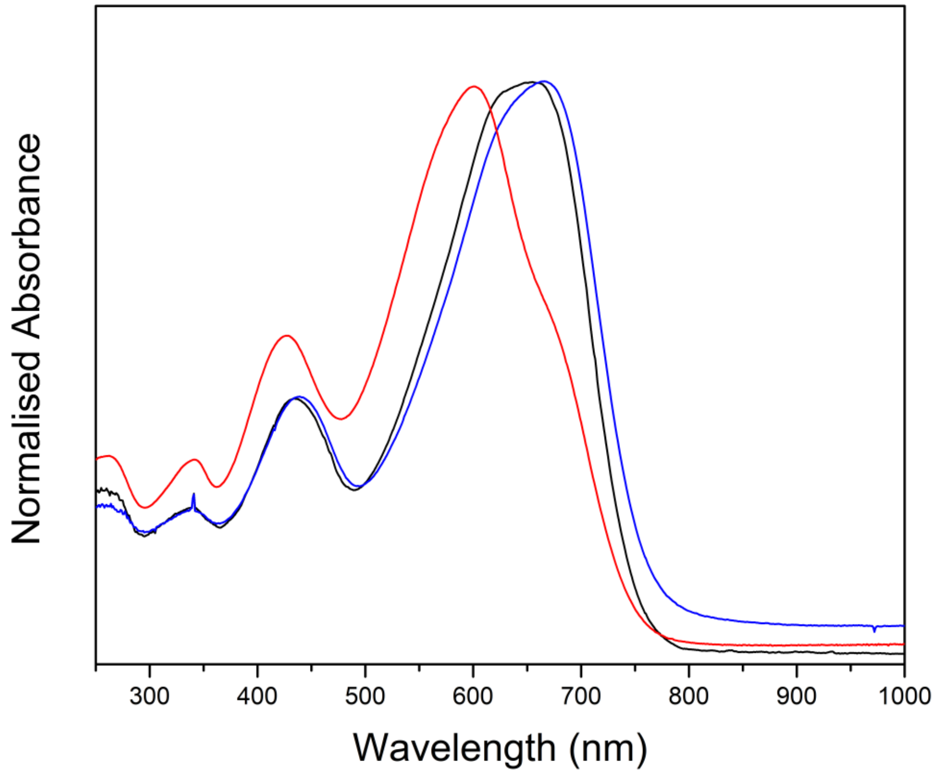

Figure S20: Solution state UV/Vis spectra of **PgBT(F)2gTT** (black), **PgBT(Ion)2gTT** (blue) and **PgBT(TriEG)2gTT** (red) in $CHCl_3$.

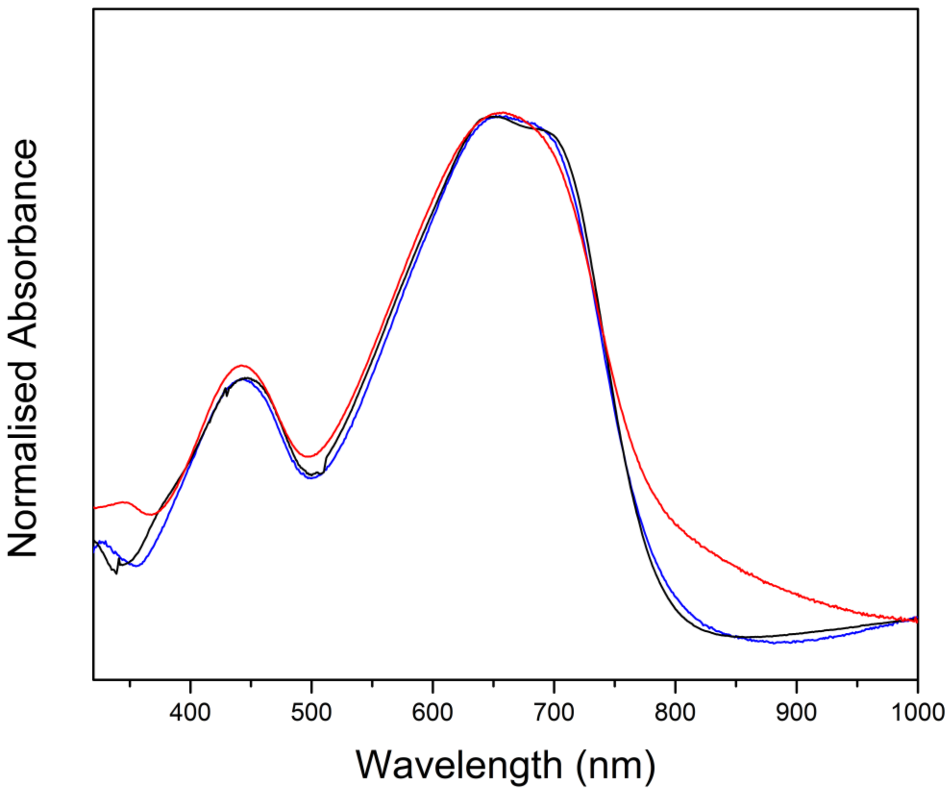

Figure S21: Solid state UV/Vis spectra of **PgBT(F)2gTT** (black), **PgBT(Ion)2gTT** (blue) and **PgBT(TriEG)2gTT** (red) thin films.

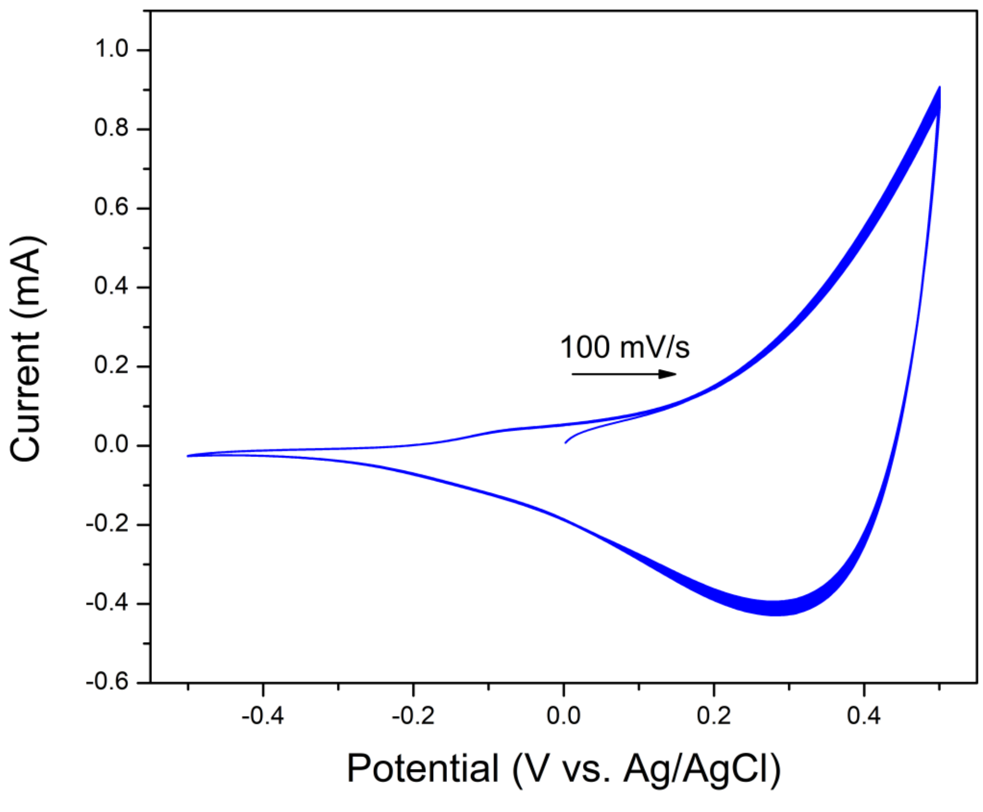

Figure S22: 100 scan cycling CV of a **PgBT(Ion)2gTT** thin film in 0.1 M KCl/H_2O. Arrow indicates scan direction.

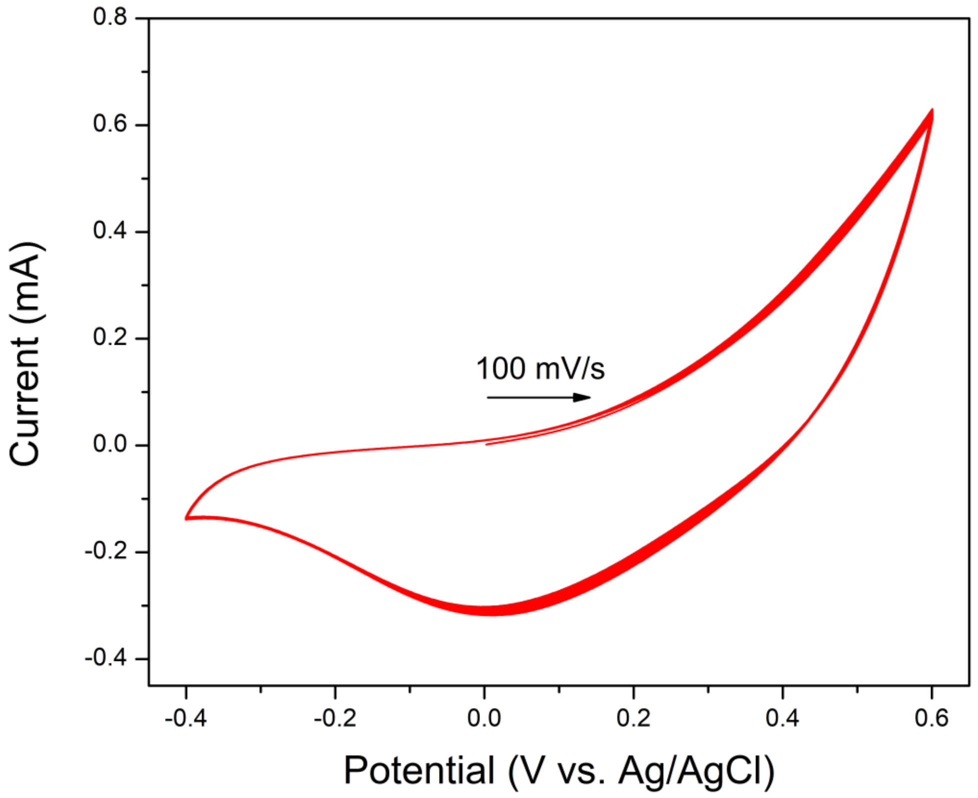

Figure S23: 100 scan cycling CV of a **PgBT(TriEG)2gTT** thin film in 0.1 M KCl/H_2O. Arrow indicates scan direction.

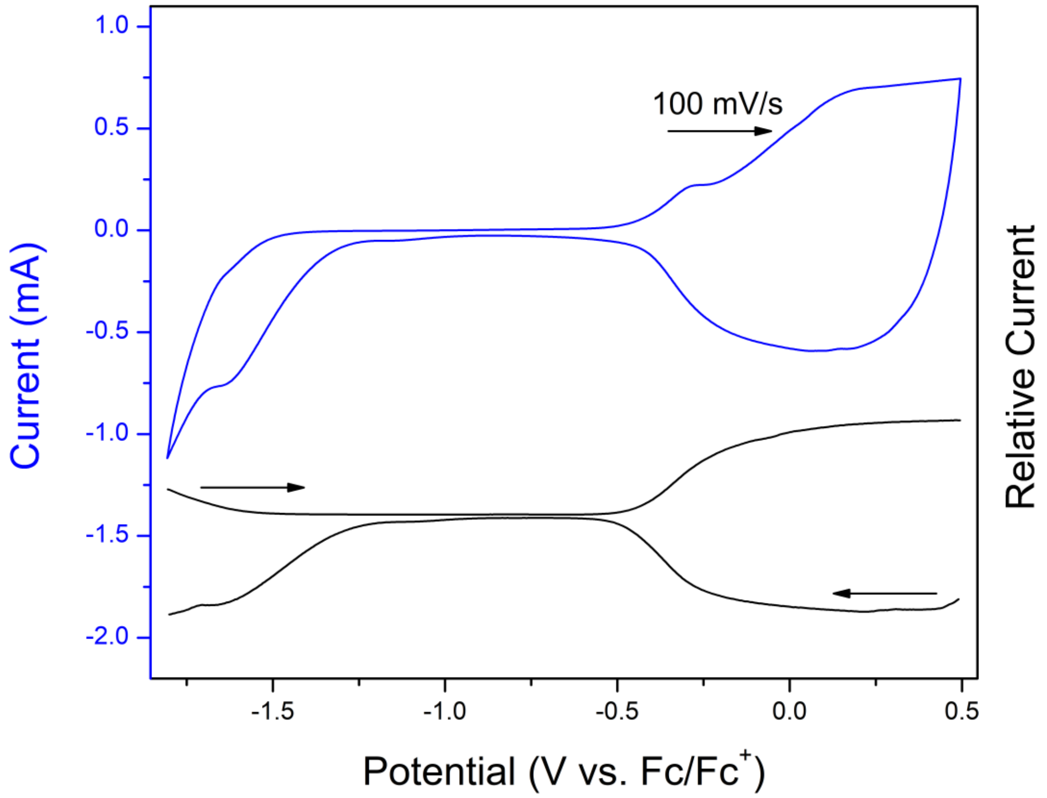

Figure S24: Solid state thin film electrochemistry of **PgBT(Ion)2gTT** in 0.1 M [*n*-Bu_4N]PF_6/MeCN showing CV (blue) and SQW (black). Arrows indicate scan direction.

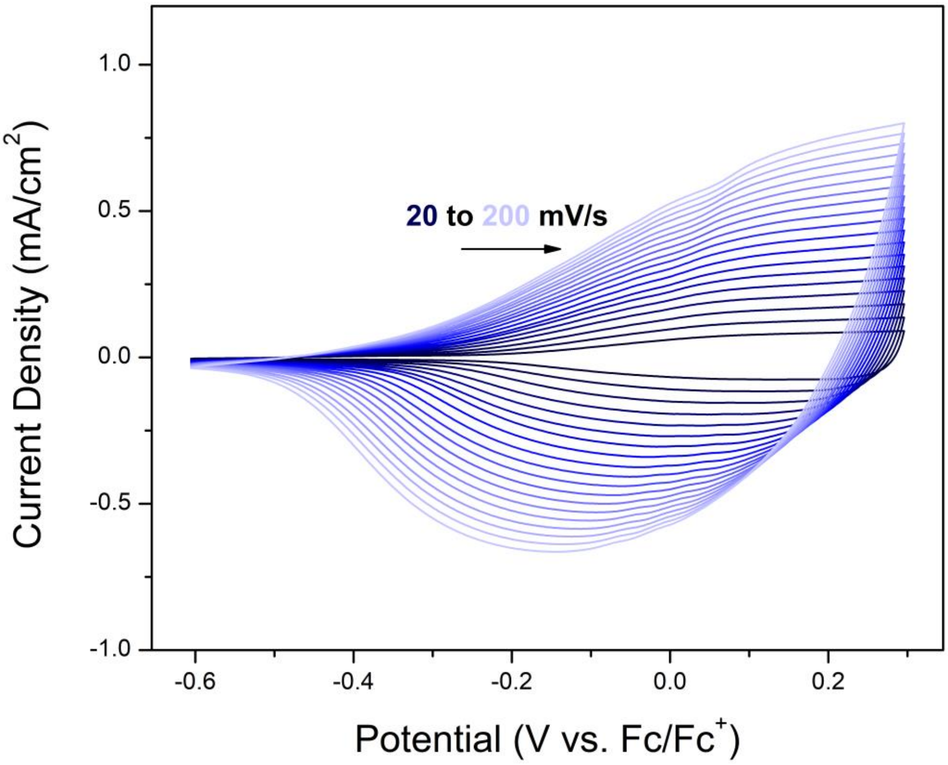

Figure S25: Solid state thin film CV of **PgBT(Ion)2gTT** in 0.1 M [*n*-Bu_4N]PF_6/MeCN at different scan rates ranging from 20 to 200 mV/s (in 10 mV/s intervals). Arrow indicates scan direction.

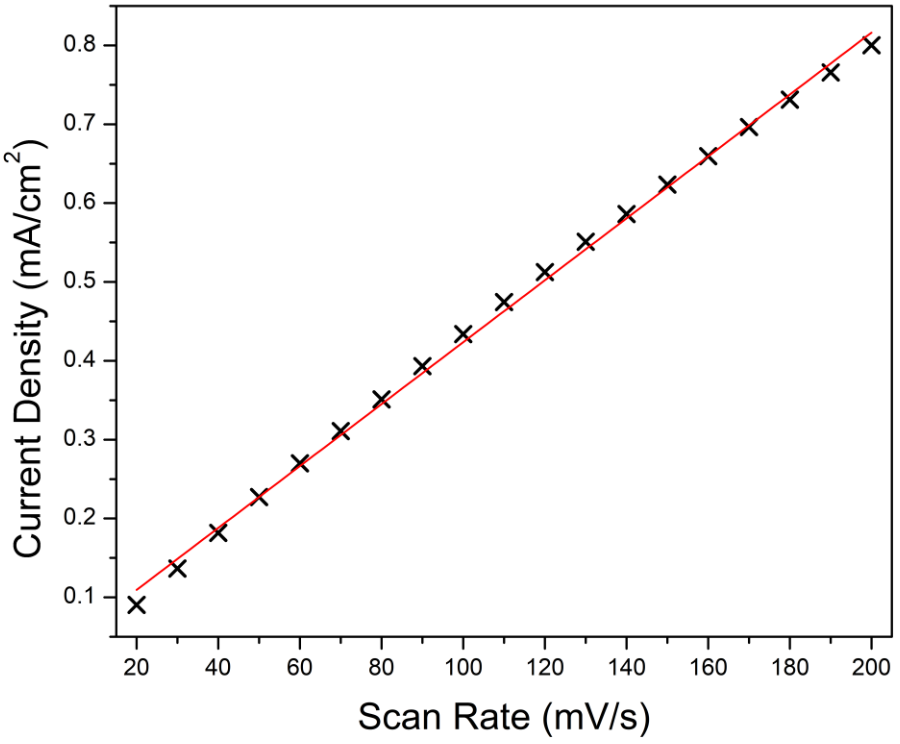

Figure S26: Plot of peak currents at 0.3 V vs. Fc/Fc^+ against their corresponding scan rates, for **PgBT(Ion)2gTT** scan rate dependence CV data presented in Figure S22. A linear correlation was observed and confirmed by regression analysis (red, R^2 = 0.99), suggesting a surface confined electrochemical process, which is explained by the larger counterion sizes of [*n*-Bu_4N]PF_6.

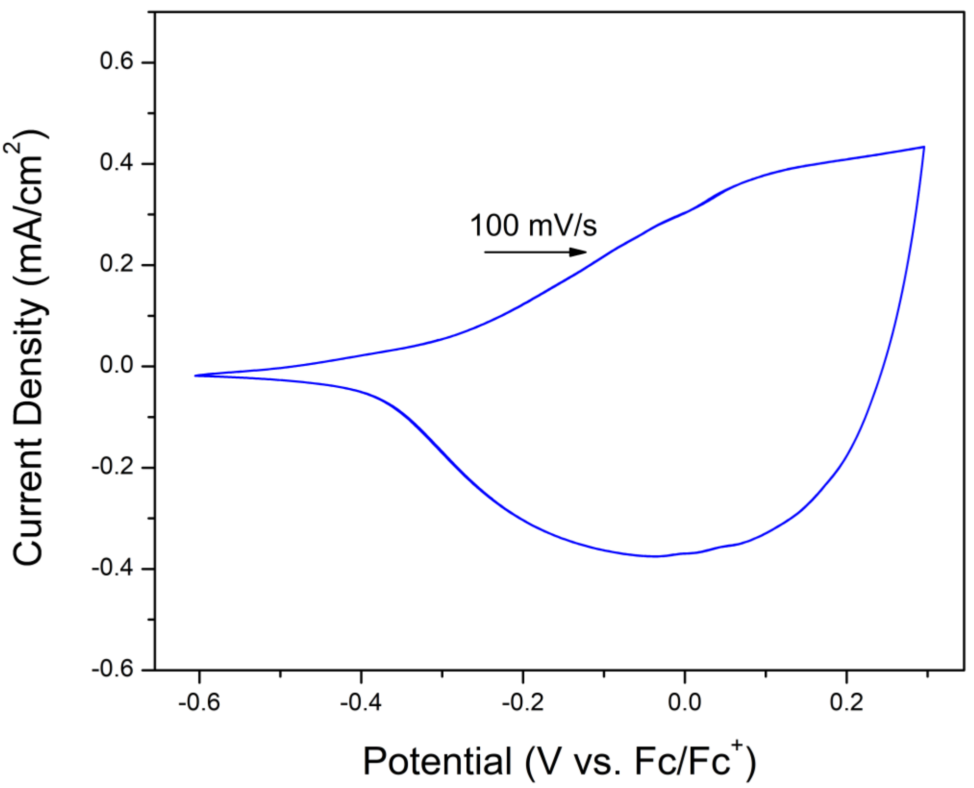

Figure S27: 12 scan cycling CV of a **PgBT(Ion)2gTT** thin film in 0.1 M [*n*-Bu_4N]PF_6/MeCN. Arrow indicates scan direction.

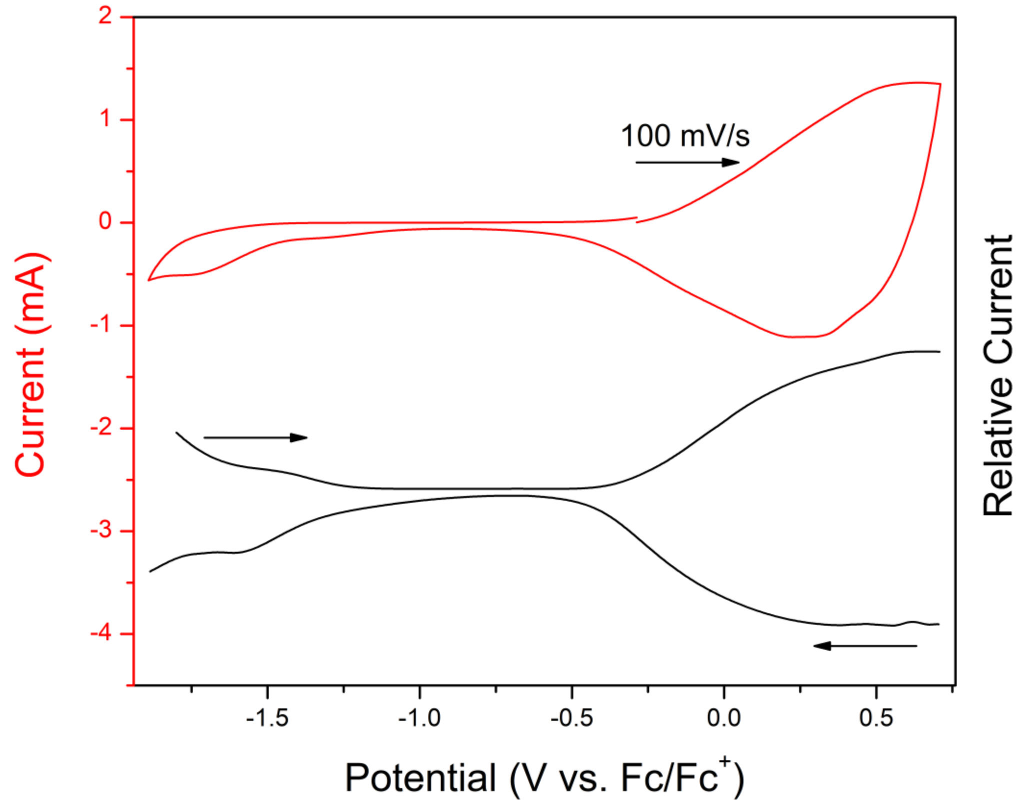

Figure S28: Solid state thin film electrochemistry of **PgBT(TriEG)2gTT** in 0.1 M [*n*-Bu_4N]PF_6/MeCN showing CV (red) and SQW (black). Arrows indicate scan direction.

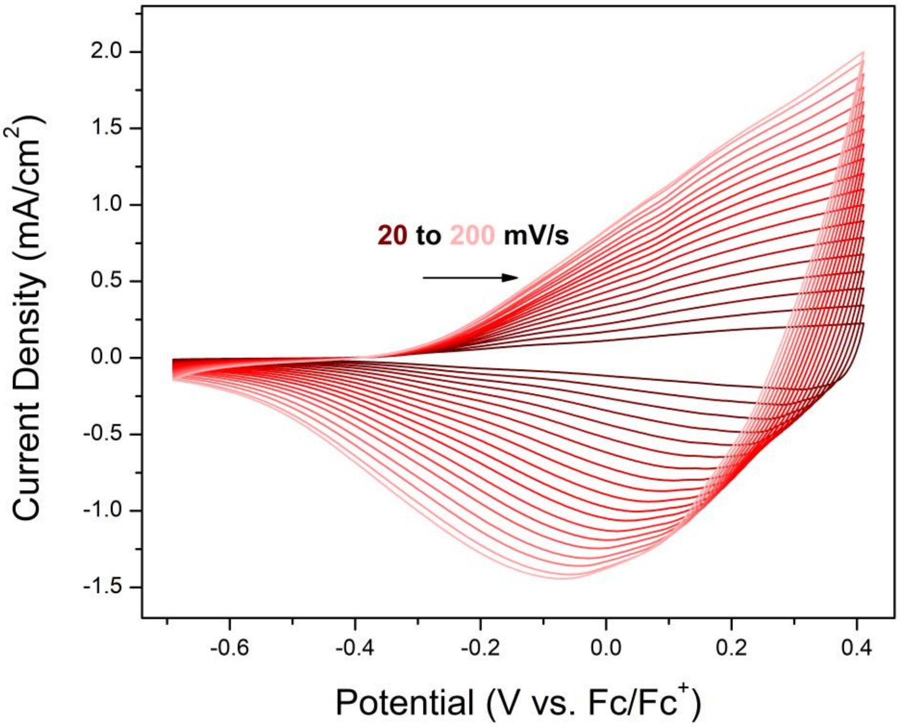

Figure S29: Solid state thin film CV of **PgBT(TriEG)2gTT** in 0.1 M [*n*-Bu_4N]PF_6/MeCN at different scan rates ranging from 20 to 200 mV/s (in 10 mV/s intervals). Arrow indicates scan direction.

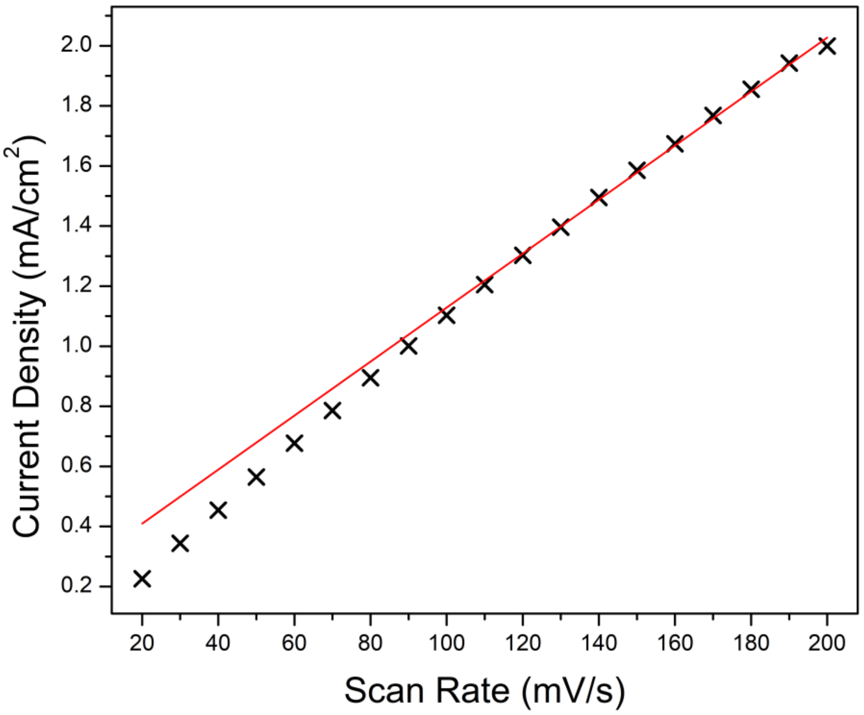

Figure S30: Plot of peak currents at 0.4 V vs. Fc/Fc$^+$ against their corresponding scan rates, for **PgBT(TriEG)2gTT** scan rate dependence CV data presented in Figure S26. For data at scan rates above 120 mV/s, a linear correlation was observed and confirmed by regression analysis (red, R^2 = 0.99), implying surface confined electrochemical oxidation, that tapers to take on diffusion limited characteristics at lower scan rates. The inhomogeneous penetration of counterions into the thin film of **PgBT(TriEG)2gTT**, particularly at higher scan rates, can be attributed to the larger ionic sizes of [*n*-Bu_4N]PF_6.

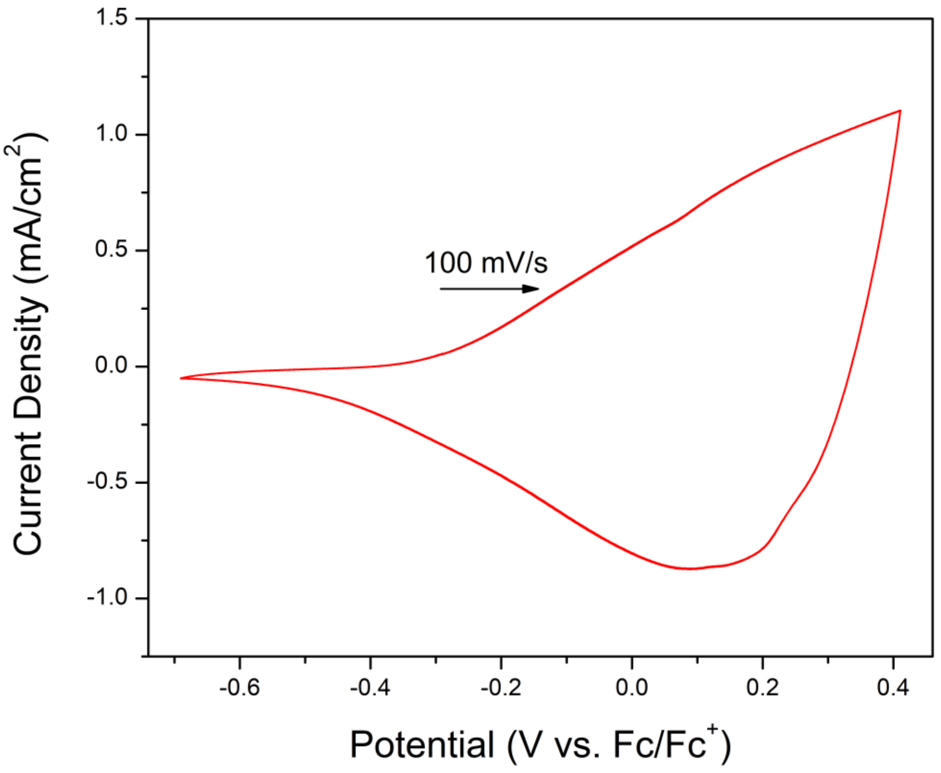

Figure S31: 12 scan cycling CV of **PgBT(TriEG)2gTT** thin film in 0.1 M [*n*-Bu_4N]PF_6/MeCN. Arrow indicates scan direction.

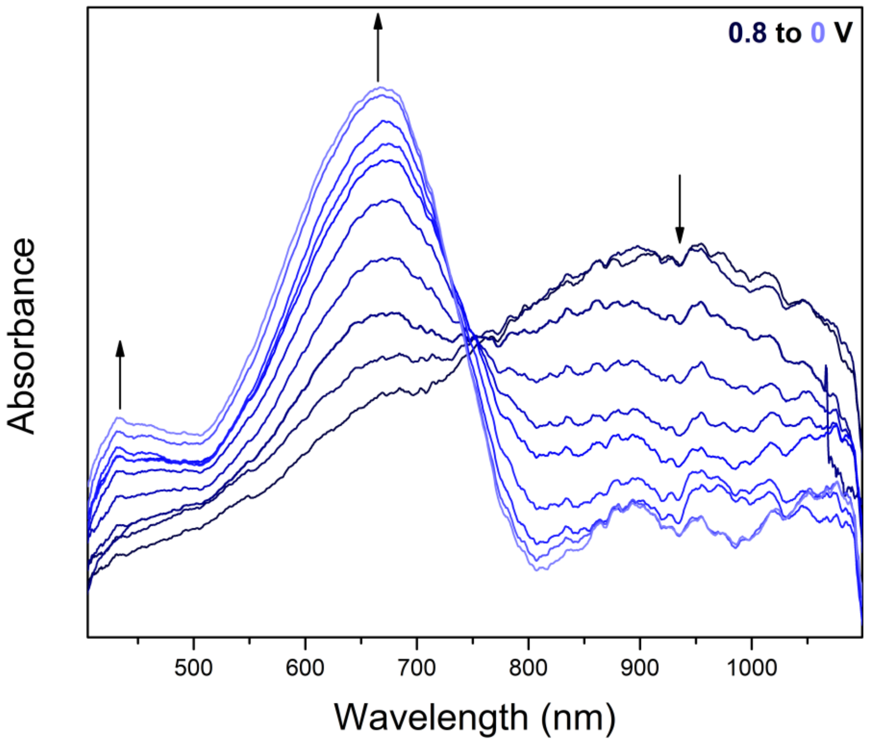

Figure S32: Solid state UV/Vis spectroelectrochemistry of **PgBT(Ion)2gTT** in 0.1 M KCl/H_2O at an initial applied potential of 0.8 V vs. Ag/AgCl, and upon returning the applied potential to 0 V in 0.1 V increments, to demonstrate electrochemical reversibility. Arrows indicate spectral changes.

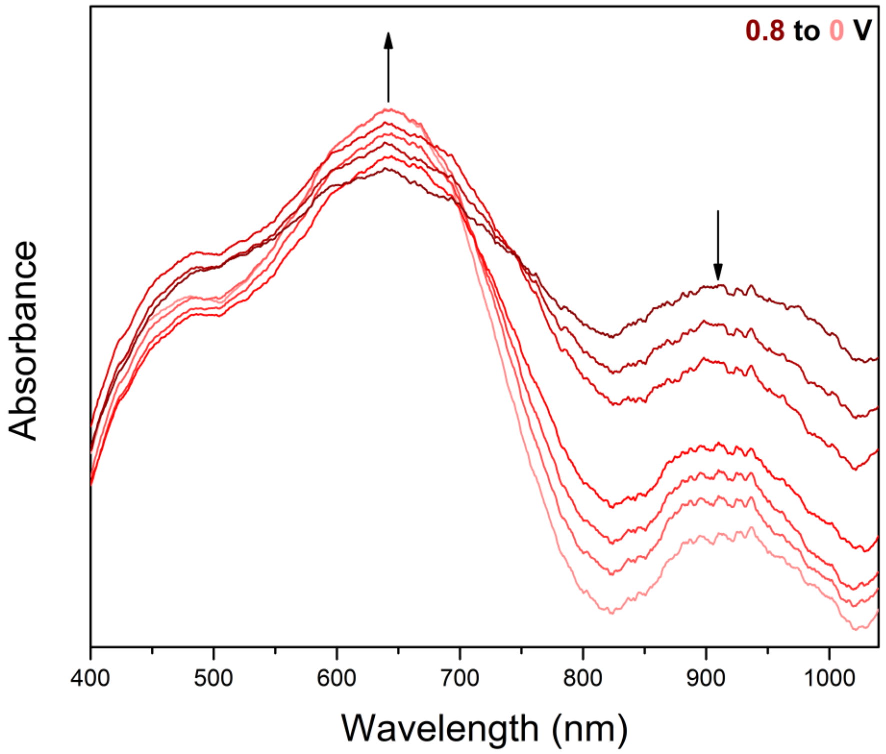

Figure S33: Solid state UV/Vis spectroelectrochemistry of **PgBT(TriEG)2gTT** in 0.1 M KCl/H_2O at an initial applied potential of 0.8 V vs. Ag/AgCl, and upon returning the applied potential to 0 V in 0.1 V increments, to demonstrate electrochemical reversibility. Arrows indicate spectral changes.

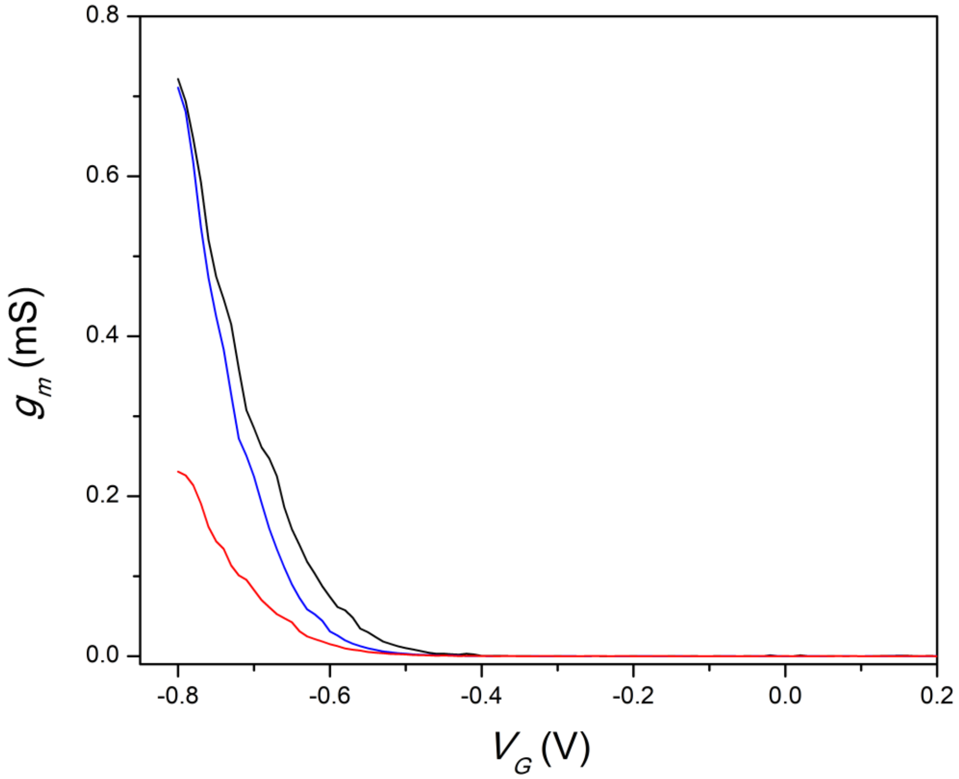

Figure S34: OECT transconductance of **PgBT(F)2gTT** (black), as compared to that of **PgBT(Ion)2gTT** (blue) and **PgBT(TriEG)2gTT** (red), at V_D = -0.60 V. Data collected with the current device configuration for comparison.

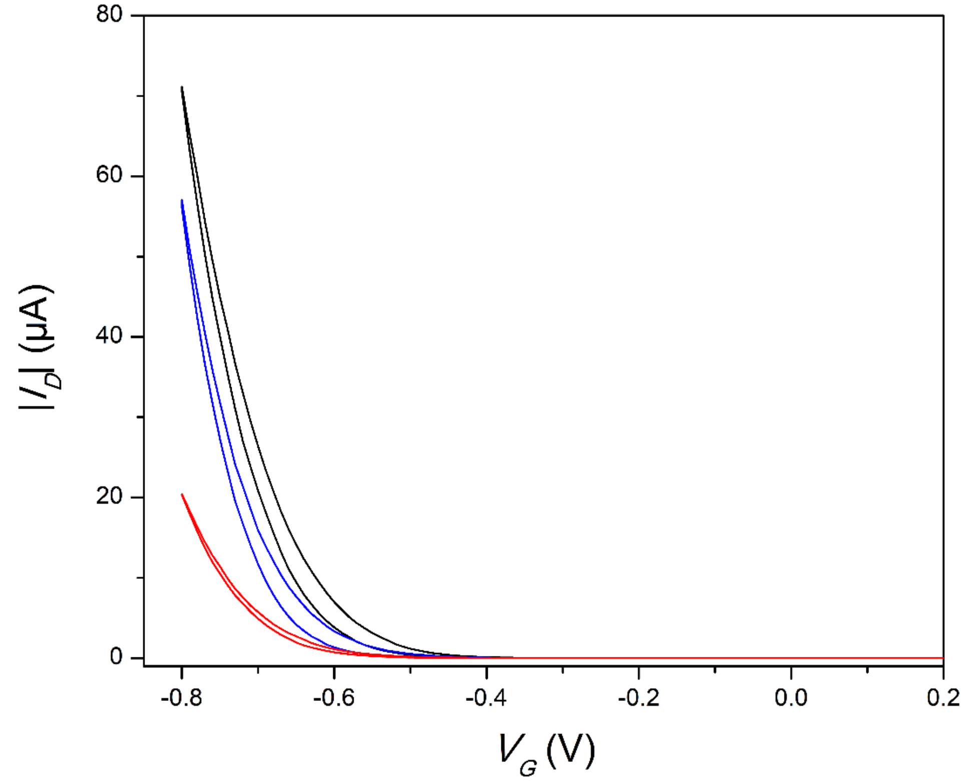

Figure S35: OECT transfer curve of **PgBT(F)2gTT** (black), as compared to that of **PgBT(Ion)2gTT** (blue) and **PgBT(TriEG)2gTT** (red), at V_D = -0.60 V. Data collected with the current device configuration for comparison.

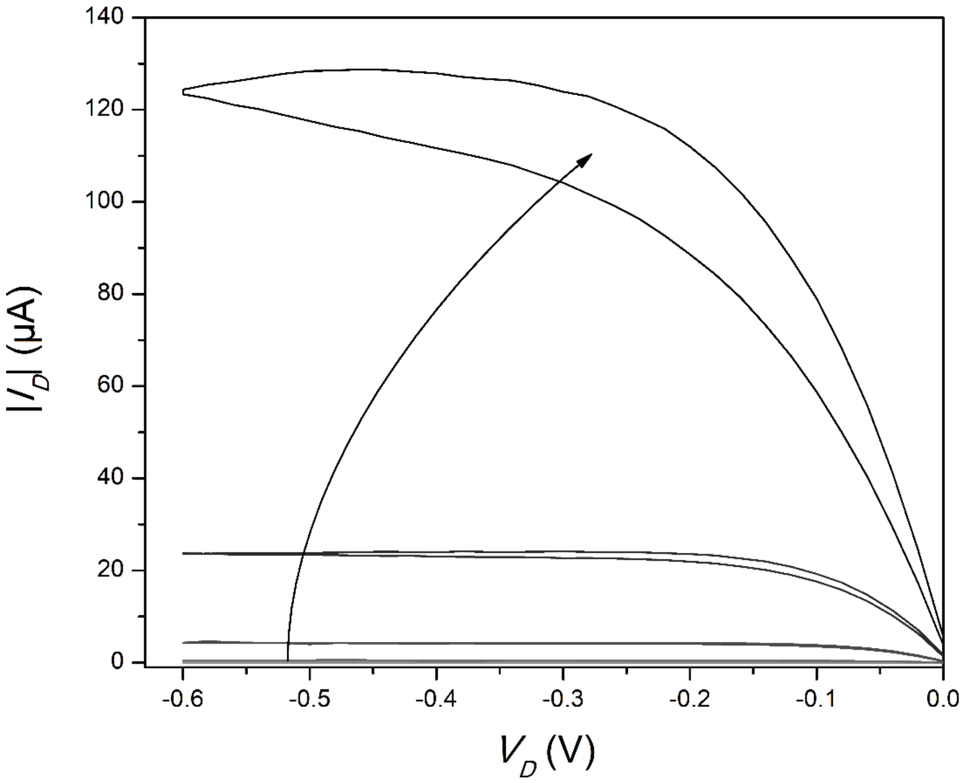

Figure S36: OECT output curves of **PgBT(F)2gTT** at stepped V_G from 0 to -0.8 in 0.1 V intervals (arrow indicating data at increased V_G). Data collected with the current device configuration for comparison.

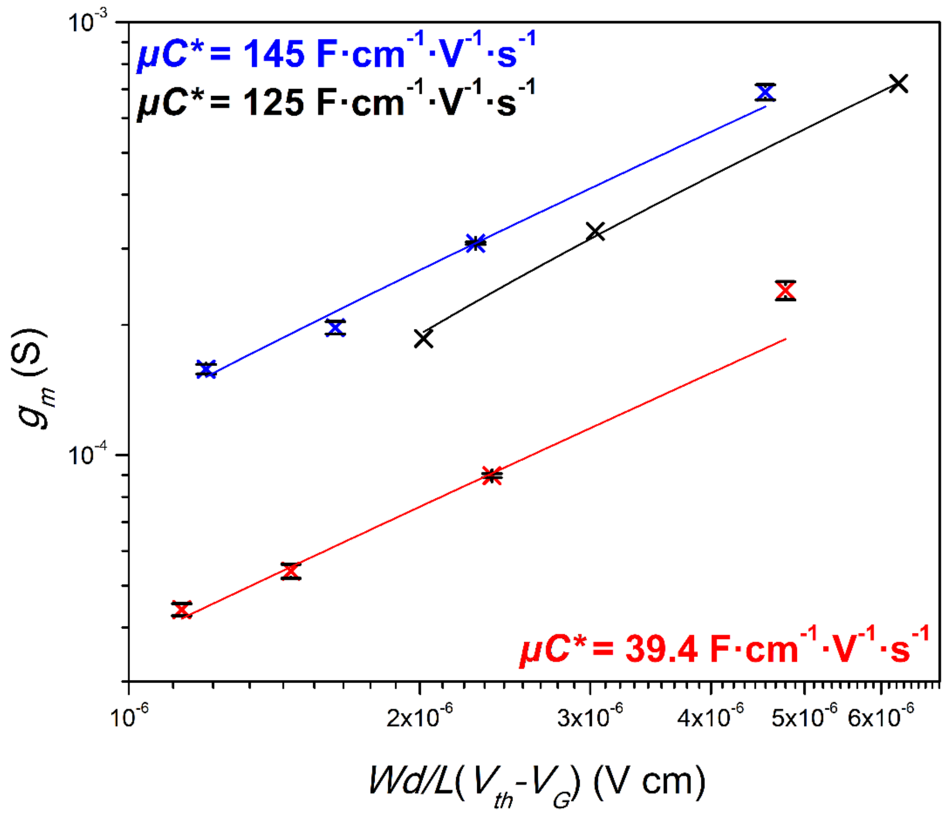

Figure S37: OECT μC^* extraction plot of transconductance against channel dimensions and operational parameters for **PgBT(F)2gTT** (black), as compared to that of **PgBT(Ion)2gTT** (blue) and **PgBT(TriEG)2gTT** (red), with linear regression analysis in respective colours. Recollected data using current device configuration for comparison.

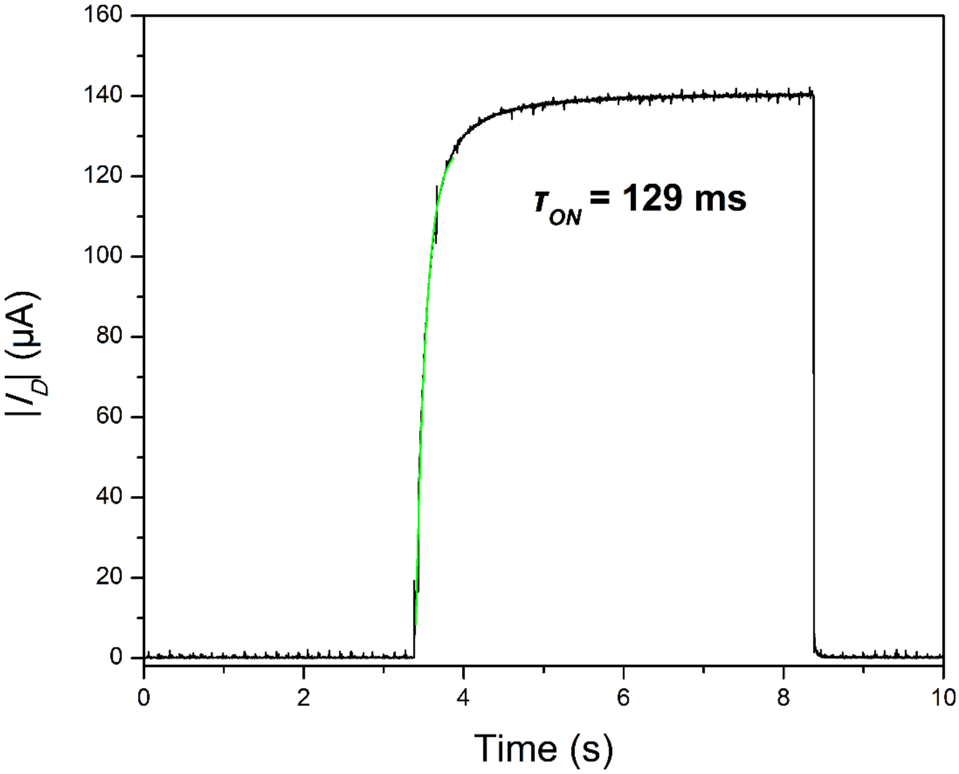

Figure S38: Transient response of the **PgBT(F)2gTT** OECTs fabricated in this study (exponential decay function to obtain τ_{ON} = 129 ms in green).

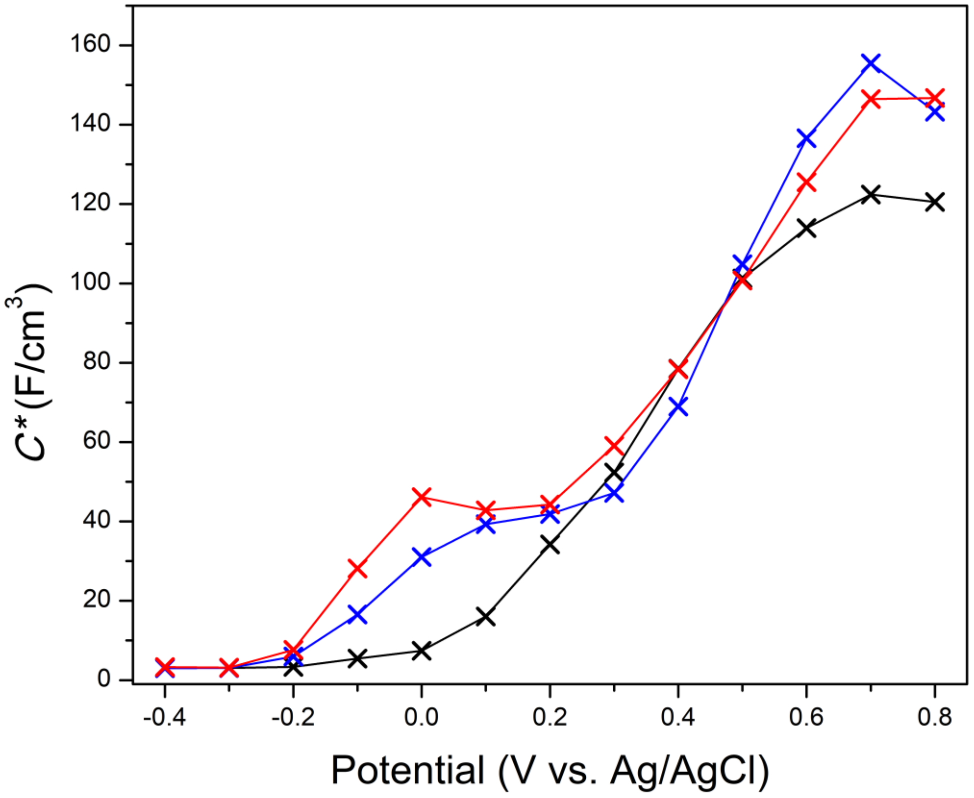

Figure S39: Volumetric capacitance of **PgBT(F)2gTT** (black), **PgBT(Ion)2gTT** (blue) and **PgBT(TriEG)2gTT** (red) measured by EIS.

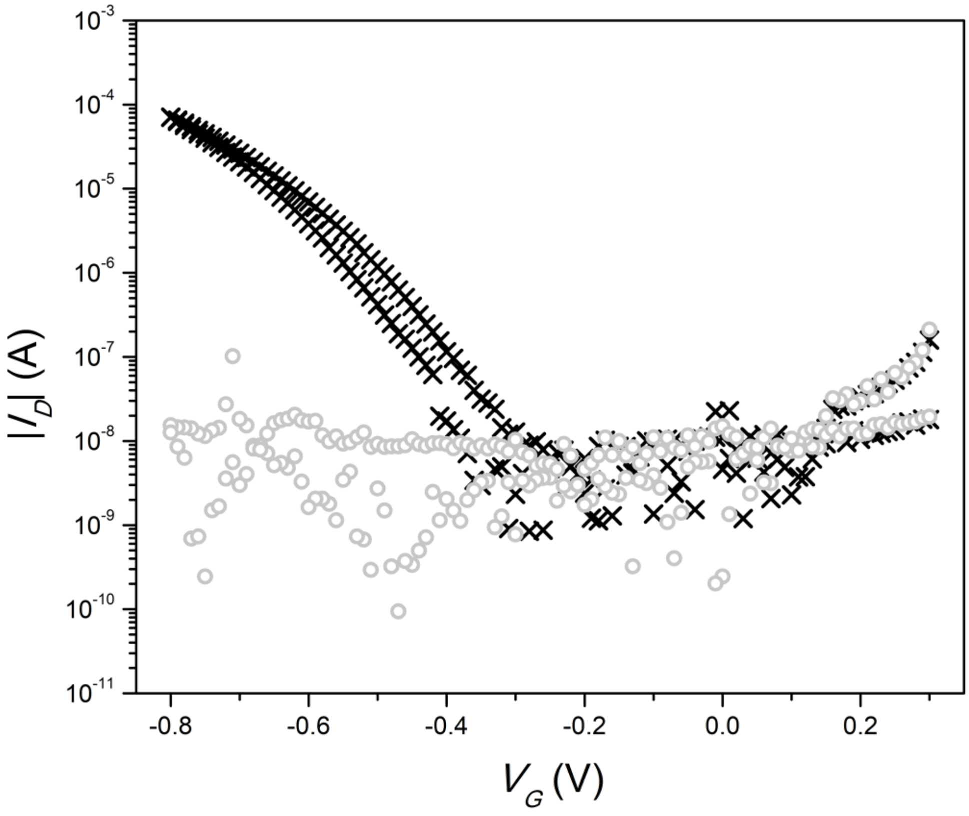

Figure S40: Log scale OECT transfer curve of **PgBT(F)2gTT** (black) with gate leakage currents (light grey).

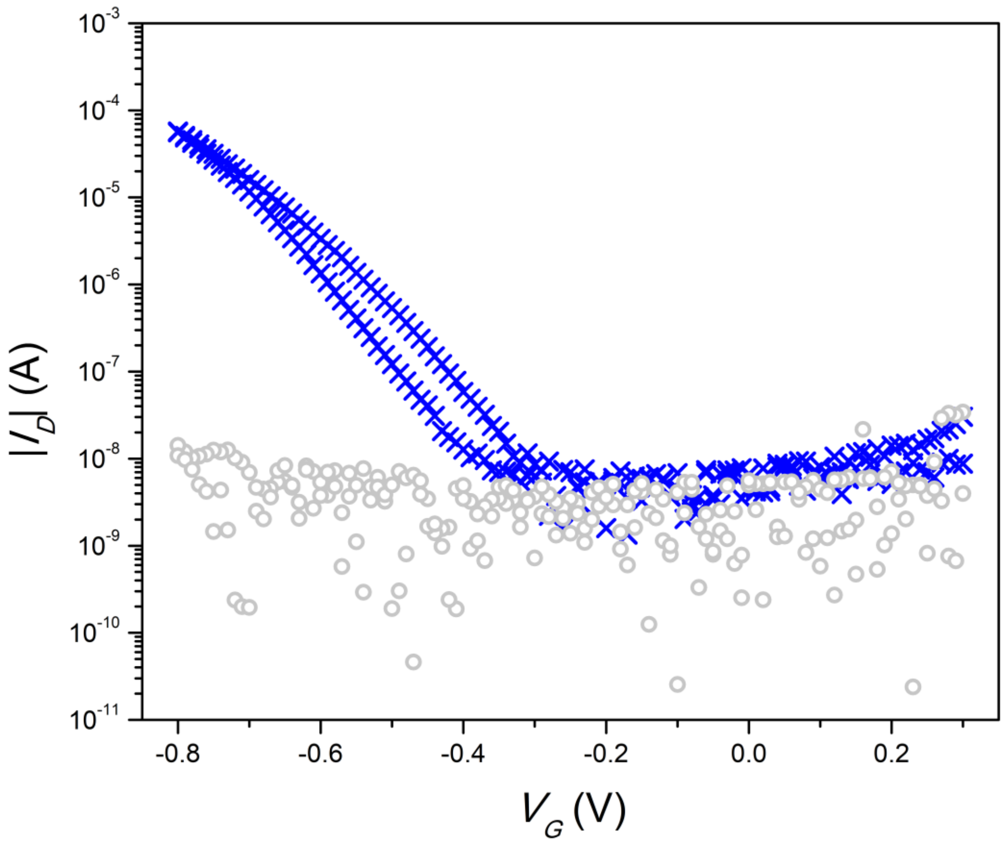

Figure S41: Log scale OECT transfer curve of **PgBT(Ion)2gTT** (blue) with gate leakage currents (light grey).

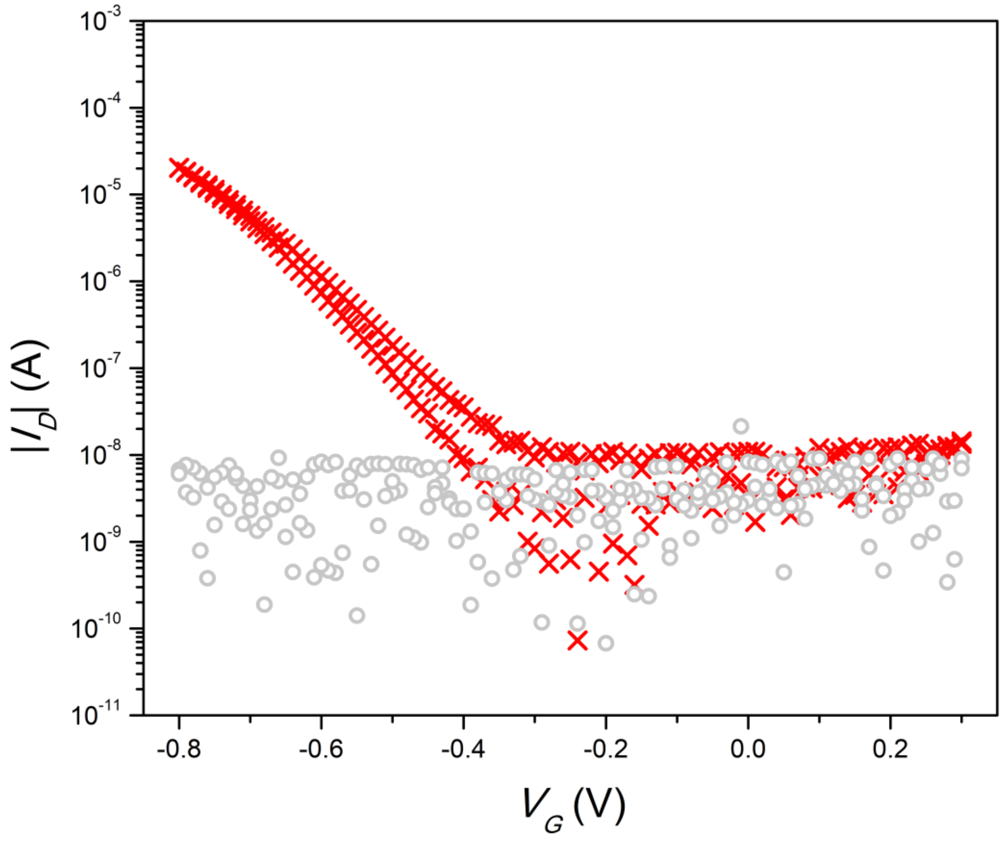

Figure S42: Log scale OECT transfer curve of **PgBT(TriEG)2gTT** (red) with gate leakage currents (light grey).

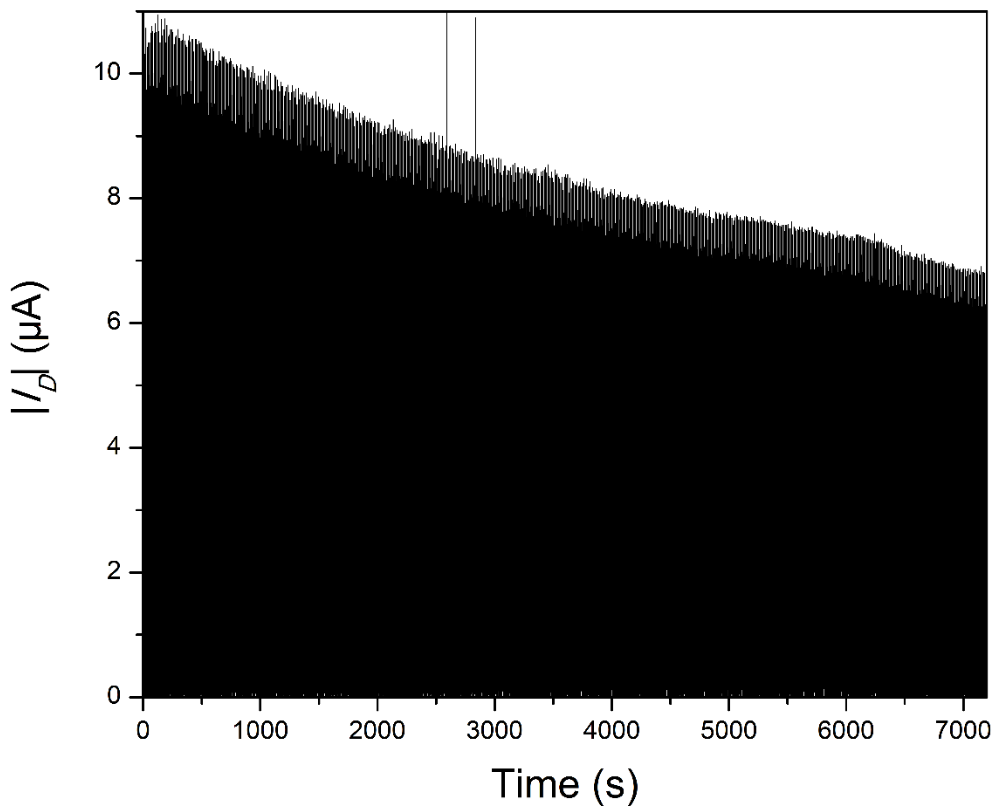

Figure S43: Long term OECT cycling stability of **PgBT(F)2gTT**.

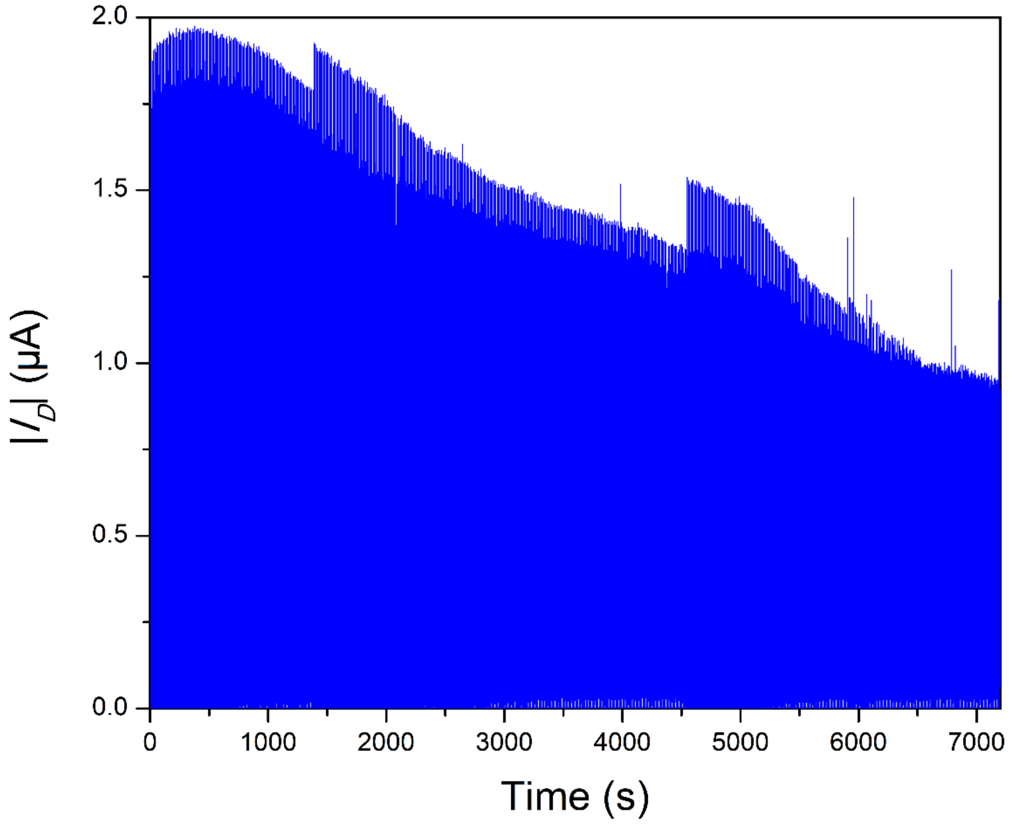

Figure S44: Long term OECT cycling stability of **PgBT(Ion)2gTT**.

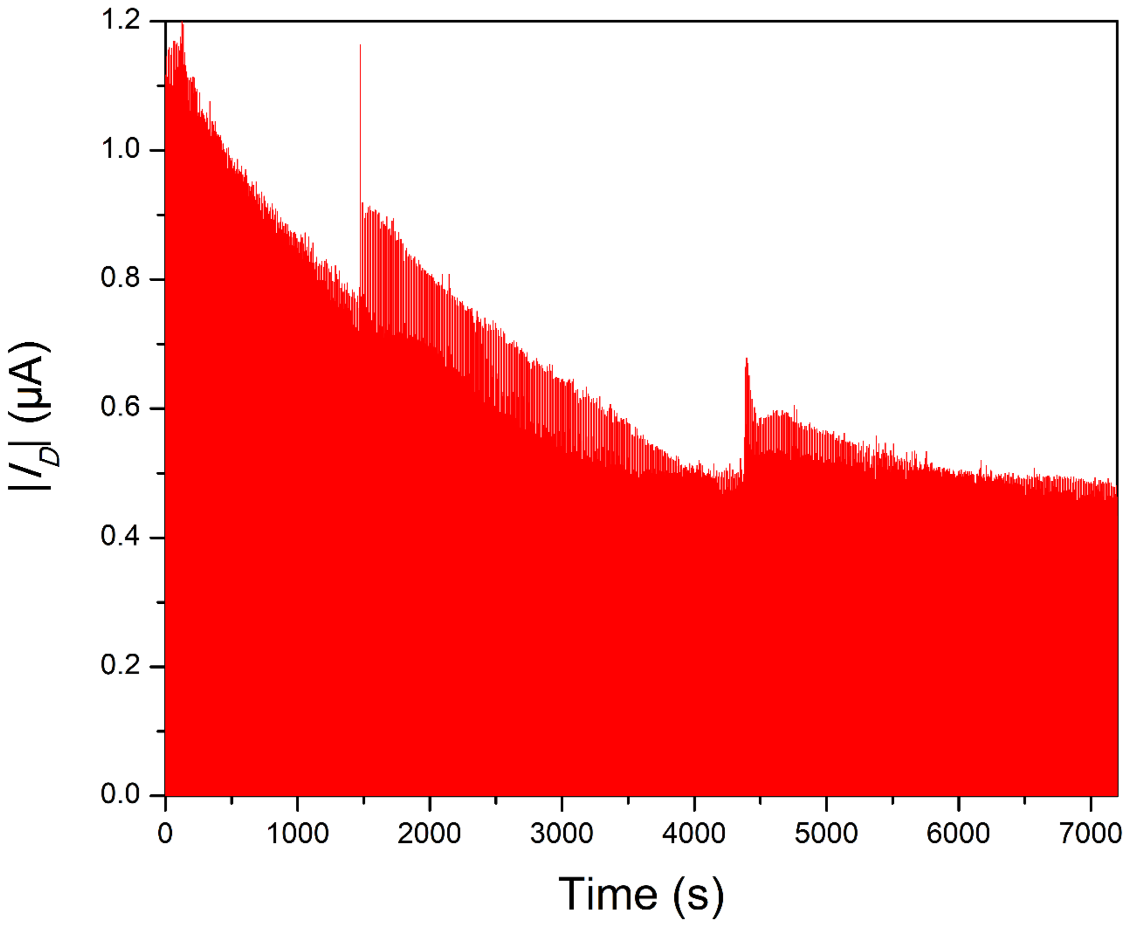

Figure S45: Long term OECT cycling stability of **PgBT(TriEG)2gTT**.

Table S1: Channel geometry normalized transconductance for **PgBT(Ion)2gTT**, **PgBT(TriEG)2gTT** and **PgBT(F)2gTT** OECTs, as compared against the performance of other recently published D-A *p*-type OECT materials.

**Polymer**	d [a] (nm)	V_{Th} [b] (V)	I_{ON}/I_{OFF} [c]	g_m [d] (mS)	$g_{m,norm}$ [e] ($S \cdot cm^{-1}$)	μC^* ($F \cdot cm^{-1} \cdot V^{-1} \cdot s^{-1}$)	C^* [f] ($F \cdot cm^{-3}$, EIS)	μ [g] ($cm^2 \cdot V^{-1} \cdot s^{-1}$)	τ_{on} [h] (ms)	
**PgBT(Ion)2gTT**	61.31 ± 3.64	-0.61	10^4	0.69 ± 0.02	28.13	145.33	143	1.02	98	*This work*
**PgBT(TriEG)2gTT**	59.72 ± 4.21	-0.60	10^3	0.24 ± 0.01	10.04	39.37	147	0.26	32	*This work*
**PgBT(F)2gTT**	60.47 ± 5.50	-0.57	10^4	0.59 ± 0.08	24.39	125.43	121	1.03	129	*This work*
**p(gDPPT2)**	90	-0.52	10^5	6.3	63	342	196	1.55	-	Ref [2]
**p(gPyDPPMeOT2)**	120	-	10^5	2.34	19.5	1.8	60	0.03	0.77	Ref [3]
**p(g1T2-g5T2)**	65	0.1	10^5	10.2	15.9	496	133	2.61	-	Ref [4]
**PTDPP-DT**	42	−0.93	10^5	7.2	19	149	123	1.1	-	Ref [5]
**P(gDTDPPbis-EDOT)**	289	-0.06	10^4	0.21	3.63	16.5	189	0.087	22	Ref [6]
**P(bgDPP-MeOT2)**	64	-0.33	10^5	5.33	83.1	195	120.0	1.63	0.516	Ref [7]
**TDPP-gTVT**	73	-0.36	10^5	3.48	47.8	205.2	173.5	1.1	7.3	Ref [8]

[a] Channel thickness. [b] Threshold voltage. [c] ON/OFF ratio. [d] Transconductance. [e] Peak transconductance normalized by channel geometry ($W \cdot d \cdot L^{-1}$). [f] Volumetric capacitance; measured by electrochemical impedance spectroscopy using a conventional 3 electrode system. [g] Charge mobility calculated from figure-of-merit (μC^*) and volumetric capacitance (C^*). [h] Transient response.

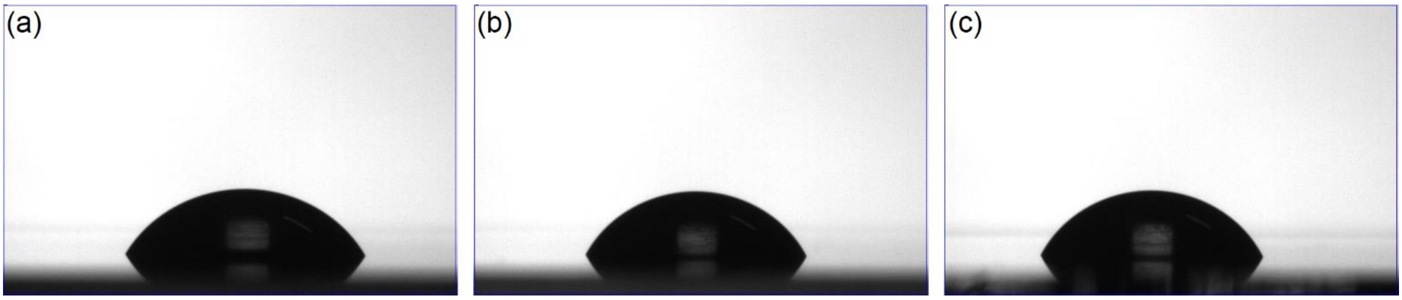

Figure S46: Water contact angle photographs for (a) **PgBT(F)2gTT**, (b) **PgBT(Ion)2gTT** and (c) **PgBT(TriEG)2gTT**.

Table S2: Water contact angle measurements for **PgBT(F)2gTT**, **PgBT(Ion)2gTT** and **PgBT(TriEG)2gTT**.

**Polymer**	*Contact Angle (°)*
**PgBT(F)2gTT**	58.67 ± 0.76
**PgBT(Ion)2gTT**	61.27 ± 1.50
**PgBT(TriEG)2gTT**	60.80 ± 0.32

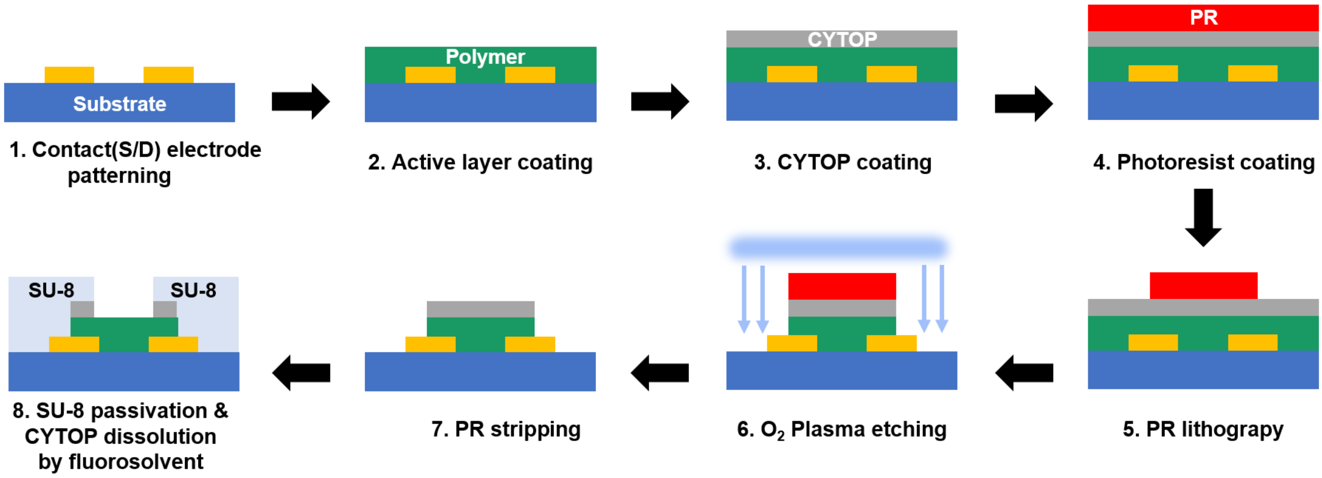

Scheme S1: Summary of the OECT fabrication procedure.

REFERENCES

1. B. Ding, G. Kim, Y. Kim, F.D. Eisner, E. Gutiérrez-Fernández, J. Martín, M-H. Yoon, M. Heeney, *Angew. Chem. Int. Ed.* **2021**, *60*, 19679.
2. M. Moser, A. Savva, K. Thorley, B.D. Paulsen, T.C. Hidalgo, D. Ohayon, H. Chen, A. Giovannitti, A. Marks, N. Gasparini, A. Wadsworth, J. Rivnay, S. Inal, I. McCulloch, *Angew. Chem. Int. Ed.* **2021**, *60*, 7777.
3. A. Giovannitti, R.B. Rashid, Q. Thiburce, B.D. Paulsen, C. Cendra, K. Thorley, D. Moia, J.T. Mefford, D. Hanifi, W. Du, M. Moser, A. Salleo, J. Nelson, I. McCulloch, J. Rivnay, *Adv. Mater.* **2020**, *32*, 1908047.

4. M. Moser, T.C. Hidalgo, J. Surgailis, J. Gladsich, S. Ghosh, R. Sheelamanthula, Q. Thiburce, A. Giovannitti, A. Salleo, N. Gasparini, A. Wadsworth, I. Zozoulenko, M. Berggren, E. Stavrinidou, S. Inal, I. McCulloch, *Adv. Mater.* **2020**, *32*, 2002748.
5. X. Wu, Q. Liu, A. Surrendran, S.E. Bottle, P. Sonar, W.L. Leong, *Adv. Electron. Mater.* **2021**, *7,* 2000701.
6. N. Wang, L. Xie, H. Ling, V. Piradi, L. Li, X. Wang, X. Zhu, F. Yan, *J. Mater. Chem. C* **2021**, *9*, 4260.
7. H. Jia, Z. Huang, P. Li, S. Zhang, Y. Wang, J.-Y. Wang, X. Gu, T. Lei, *J. Mater. Chem. C* **2021**, *9*, 4927.
8. Y. Wang, A. Hamidi-Sakr, J. Surgailis, Y. Zhou, H. Liao, J. Chen, G. Zhu, Z. Li, S. Inal, W. Yue, *J. Mater. Chem. C* **2021**, *9*, 13338.
